# Quantifying the contribution of triglycerides to metabolic resilience through the mixed meal model

**DOI:** 10.1016/j.isci.2022.105206

**Published:** 2022-10-03

**Authors:** Shauna D. O’Donovan, Balázs Erdős, Doris M. Jacobs, Anne J. Wanders, E. Louise Thomas, Jimmy D. Bell, Milena Rundle, Gary Frost, Ilja C.W. Arts, Lydia A. Afman, Natal A.W. van Riel

**Affiliations:** 1 Division of Human Nutrition and Health, Wageningen University, Wageningen, the Netherlands; 2 Department of Biomedical Engineering, Eindhoven University of Technology, Eindhoven, the Netherlands; 3 Eindhoven Artifical Intelligence Systems Institute (EAISI), Eindhoven University of Technology, Eindhoven, the Netherlands; 4 Maastricht Centre for Systems Biology (MaCSBio), Maastricht University, Maastricht, the Netherlands; 5 Unilever Global Food Innovation Centre, Bronland 14, 6708WH Wageningen, the Netherlands; 6 Research Center for Optimal Health, School of Life Sciences, University of Westminster, London, UK; 7 Division of Diabetes, Endocrinology, and Metabolism, Department of Medicine, Imperial College London, London, UK

**Keywords:** Human metabolism, Systems biology, In silico biology, Nutrition

## Abstract

Despite the pivotal role played by elevated circulating triglyceride levels in the pathophysiology of cardio-metabolic diseases many of the indices used to quantify metabolic health focus on deviations in glucose and insulin alone. We present the Mixed Meal Model, a computational model describing the systemic interplay between triglycerides, free fatty acids, glucose, and insulin. We show that the Mixed Meal Model can capture deviations in the post-meal excursions of plasma glucose, insulin, and triglyceride that are indicative of features of metabolic resilience; quantifying insulin resistance and liver fat; validated by comparison to gold-standard measures. We also demonstrate that the Mixed Meal Model is generalizable, applying it to meals with diverse macro-nutrient compositions. In this way, by coupling triglycerides to the glucose-insulin system the Mixed Meal Model provides a more holistic assessment of metabolic resilience from meal response data, quantifying pre-clinical metabolic deteriorations that drive disease development in overweight and obesity.

## Highlights

• The Mixed Meal Model describes both lipid and carbohydrate metabolism
• The Meal Model quantifies insulin resistance, β-cell functionality, and liver fat
• The Model reduces meal data to a three-dimensional measure of metabolic health
• Physiology-informed regularization produces more reliable and relevant parameters

## Graphical abstract

**Figure:**
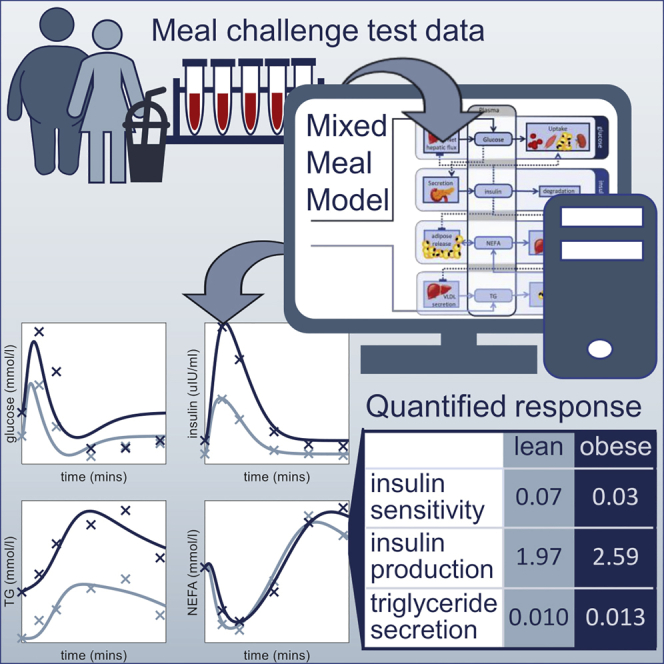

## Introduction

Health was once considered to simply be the absence of disease or injury. However, increasingly the concept of health is being re-defined as the ability of an individual to respond and adapt to physical, emotional, or social challenges, often referred to as resilience (Huber et al., 2011; Luthar et al., 2000). Metabolic resilience, generally defined as the body’s ability to recover and maintain optimal circulating levels of nutrients in response to external stresses such as food intake, physical activity, or periods of fasting, is governed by a complex interplay between multiple tissues and organs including the brain, liver, skeletal muscle, and adipose tissue. Liberating nutrients such as glucose and non-esterified fatty acids (NEFAs) from body stores in the fasting state and quickly and effectively removing excess nutrients from the plasma following the consumption of a meal (van Ommen et al., 2014). In overweight and obesity, the excessive accumulation of adipose tissue can disrupt the delicate balance between these tissues and organs (Hill et al., 2012), leading to raised circulating levels of NEFAs and triglycerides (Lewis et al., 2002; Unger, 2003). This dyslipidemia can lead to ectopic fat deposition (Avramoglu et al., 2006; Bergman and Ader, 2000) directly contributing to the development of insulin resistance in the liver and skeletal muscle (Eckel et al., 2005; Kahn et al., 2006), potentially leading to a loss of glycemic control (Reaven, 1988). Consequently, to fully understand the pre-clinical metabolic deteriorations observed in overweight and obesity it is important to consider the role of elevated plasma triglyceride concentrations (Hassing et al., 2012; Yuan et al., 2007), in addition to the rise in glucose and insulin, in the pathophysiology of cardio-metabolic diseases, which are among the leading causes of mortality in developed countries (Bays et al., 2004; Ford, 2005; Higgins and Adeli, 2017).

In the clinic, fasting plasma glucose and triglyceride concentration or glycated hemoglobin (HbA1c), a marker of long-term glycaemic control, are used as indicators of metabolic health (American Diabetes Association, 2011; Reaven, 1988; WHO, 2006). However, the metabolic dysregulation underlying a loss of glycemic control is observable in the postprandial state long before deviations are detectable in the fasting state (van Ommen et al., 2014). Consequently, in line with the increased focus on resilience as a measure of health, challenge tests such as oral glucose tolerance tests (OGTT) or high-calorie mixed meal challenge tests (MMT) are regularly employed in research to assess the body’s capacity to clear excess nutrients such as glucose and fat from the blood (Wopereis et al., 2017). Nevertheless, how best to quantify and interpret these multivariate meal responses still presents researchers with many challenges (Vis et al., 2015).

Several simple summary measures aiming to quantify insulin resistance or beta-cell functioning from fasting and mean postprandial glucose and insulin concentrations have been proposed in the literature (Matsuda and DeFronzo, 1999). More recently, metrics capturing specific dynamic properties of the meal response curve, such as the rate of decay of the glucose curve, have been proposed to quantify tissue-specific insulin resistance (Abdul-Ghani et al., 2007). However, these indices quantify impairments in the glucose-insulin system alone and do not account for deviations in plasma triglyceride levels which may have predictive relevance, particularly as an early marker of cardio-metabolic disease risk. Moreover, the majority of these indices have only been validated on standardized 75g OGTTs and are not readily generalizable for use on complex meals or free-living conditions.

Metrics such as the incremental area under the curve, time in range, or postprandial rise in plasma concentration are regularly employed to quantify responses to complex meals (Berry et al., 2020; Vis et al., 2015; Zeevi et al., 2015). However, such measures fail to capture dynamic properties of the post-meal plasma metabolite trajectories such as peak height and time that may have clinical significance. Furthermore, these approaches analyze the response of each metabolite independently neglecting the interplay between triglycerides, glucose, and insulin. There is a need for a generalizable metric that can integrate and quantify the post-meal trajectories of multiple macro-nutrients, providing a more holistic assessment of metabolic resilience.

Physiology-based mathematical models (PBMMs) are mathematical representations of key metabolic process that underly a given biological system and have been applied to study how interactions between different metabolic species give rise to observed system behavior (Fischer, 2008). One such model proposed by Dalla Man et al. describes the dynamics between glucose and insulin under fasting and fed conditions and has been used as an alternative to animal testing when training control algorithms for insulin pump devices (Dalla Man et al., 2007, 2014; Kovatchev et al., 2009). More recently, the application of a physiology-based computational model of the glucose-insulin system to a large population of overweight and obese individuals was shown to capture features of insulin sensitivity and rate of insulin secretion from OGTT responses (Erdős et al., 2021). Although these models have been shown to capture responses to complex meals, their focus on the glucose-insulin system means that, like the simple summary measures applied to OGTTs, they fail to capture deviations in postprandial plasma triglyceride trajectories that may provide insight into processes that drive the development of cardio-metabolic diseases.

In this study, we present the Mixed Meal Model a computational model introducing the systemic postprandial interplay between triglyceride and NEFA in the glucose-insulin system with the aim of capturing the pre-clinical deteriorations in metabolic resilience that underly overweight and obesity. To ensure the Mixed Meal Model is sufficiently robust to describe responses to meals with different macro-nutrient compositions, we apply the model to meal challenge test data from two independent diet intervention studies. Moreover, we apply the Mixed Meal Model to sub-populations defined by insulin resistance status and hepatic fat accumulation to show the model can capture physiologically relevant features of metabolic resilience from meal response data. The Mixed Meal Model provides a new objective definition of metabolic resilience, reducing the multi-dimensional time series of glucose, insulin, triglycerides, and NEFA to a three-dimensional “health-space” simultaneously quantifying insulin resistance, hepatic lipid accumulation, and beta-cell functionality. In this way, the Mixed Meal Model can be used to elucidate the role of dietary lipids and dyslipidemia in the pathophysiology of cardio-metabolic diseases.

## Results

**Figure 1:**
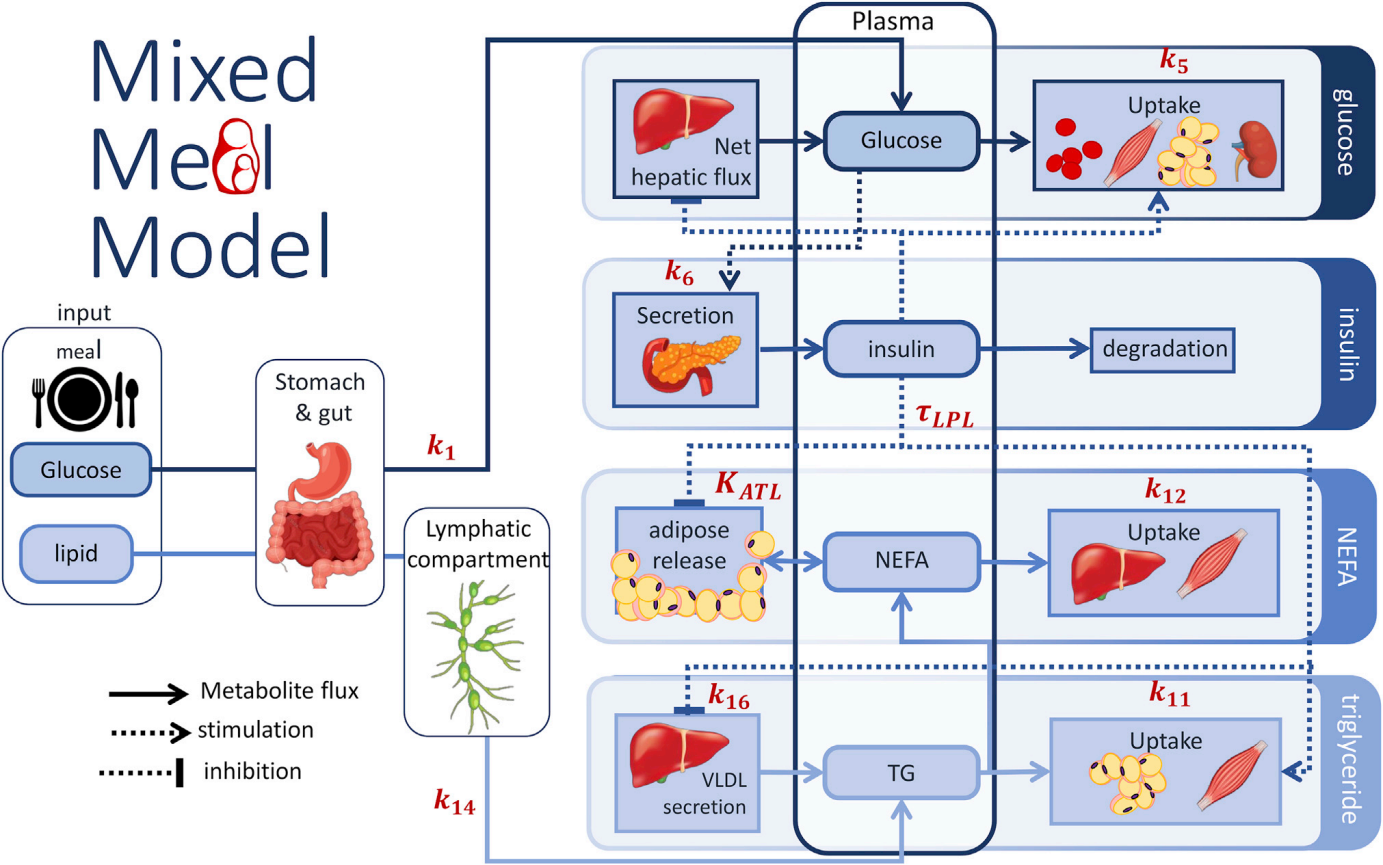
Scheme of Mixed Meal model The Mixed Meal Model describes the plasma dynamics of glucose, insulin, triglyceride (TG), and non-esterified fatty acids (NEFA). Meal-derived glucose is described as entering the plasma via the gut and endogenous glucose released into the plasma by the liver is suppressed in the postprandial state by insulin. Glucose leaves the plasma and is taken up by tissues via insulin dependent and insulin independent pathways. Insulin is produced in response to increases in plasma glucose. Meal derived triglyceride passes through a gut and lymphatic compartment before appearing in the plasma. As with glucose, endogenous triglyceride secreted into the plasma from the liver can be inhibited by increased plasma insulin. Circulating triglyceride is removed from the plasma by LPL lipolysis. The release of NEFA from the adipose tissue is inhibited by increased insulin in the postprandial state, NEFA can also spill-over into the plasma from LPL lipolysis of circulating triglyceride. Plasma NEFA is taken up into peripheral tissues at a rate proportional to the circulating NEFA concentration.

The Mixed Meal Model is a physiology-informed mathematical model describing the systemic interplay between glucose, insulin, triglycerides, and NEFA, summarized in Figure 1. Meal-derived glucose and triglyceride enter the plasma via the gut and lymphatic system respectively. Insulin is produced in response to increase in plasma glucose concentrations and acts as a master regulator in the system; the secretion of endogenously produced glucose and triglyceride by the liver is inhibited by insulin and plasma glucose is taken up into the tissues at both an insulin dependent and independent rate, hydrolysis of circulating triglyceride by lipoprotein lipase is stimulated by insulin, and the release of NEFA from the adipose tissue is inhibited by insulin. In this way, we see that insulin is the key component of metabolic resilience linking carbohydrate and lipid metabolism during the meal response. Consequently, metabolic deteriorations such as insulin resistance not only affect glucose homeostasis but also impacts lipid metabolism. The Mixed Meal Model was constructed by extending an existing model of glucose and insulin dynamics (Rozendaal et al., 2018) with terms to account for the interplay between triglycerides and non-esterified fatty acids (NEFAs). These new lipid terms were either derived from previously published models of lipid metabolism (O’Donovan et al., 2019) or formulated-based observations from lipid tracer studies in humans (Adiels et al., 2007; McQuaid et al., 2011; Ruge et al., 2009). The average meal response from the NutriTech Study (TNO, 2016) was to evaluate prospective model terms during model constructions. The generalizability of the Mixed Meal Model was then assessed by applying the model to meal response data from the MetFlex Study (Fechner et al., 2020), an independent diet intervention study. A more expansive description of the model development can be found in the STAR Methods section.

Model parameters were estimated by minimizing the error between the model simulation and measured meal response data. In addition, physiology-informed regularization, whereby the cost function used to fit the model to data was extended to include terms that penalized undesirable behaviors, such as nutrient dumping or a failure to return to the measured steady state, was introduced. Thereby guiding the parameter estimation procedure to more favorable regions of the solution space.

### Average meal responses

**Figure 2:**
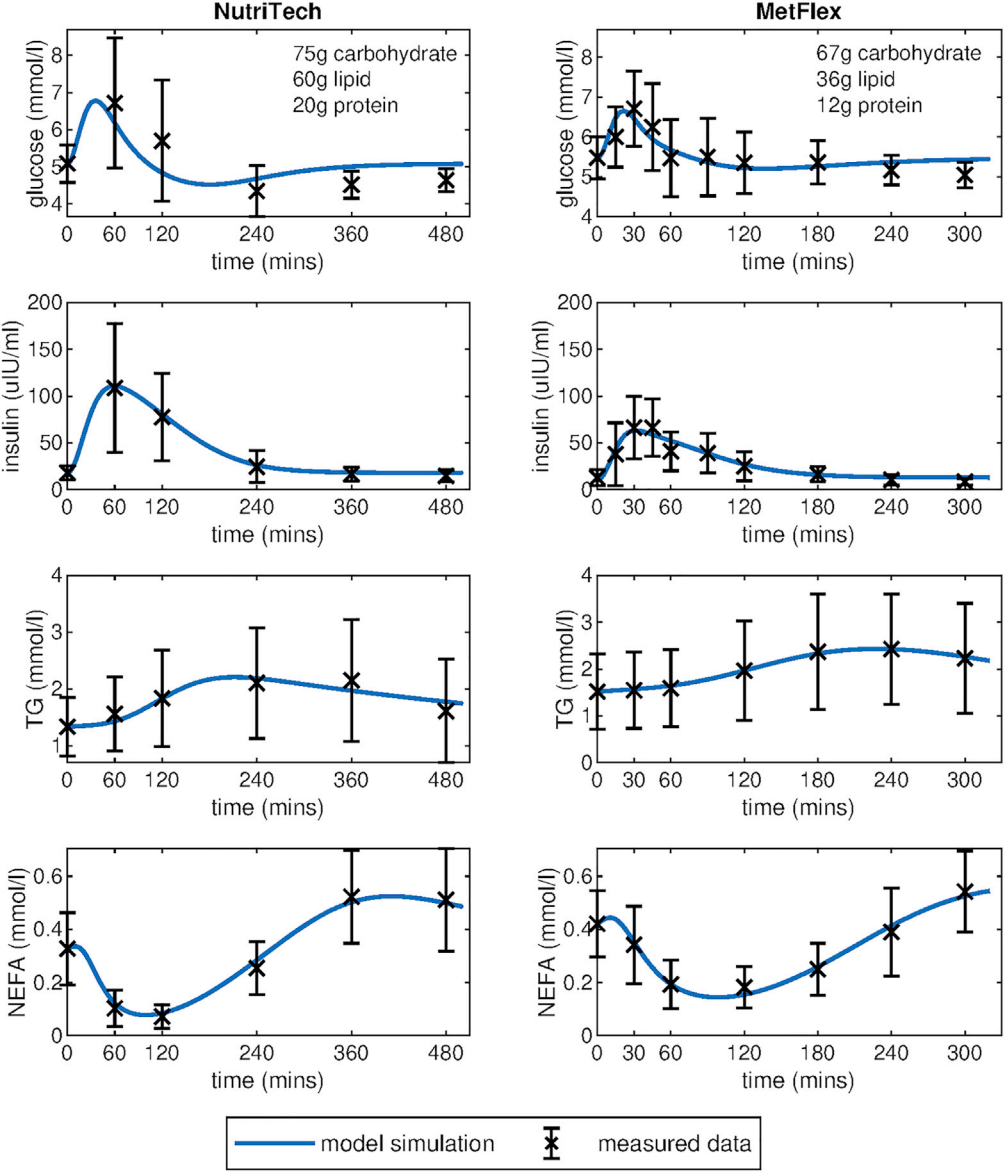
Fit of Mixed Meal model to average meal response from the NutriTech and MetFlex studies Visualization of the model fit to the average meal response of plasma glucose, insulin, triglyceride, and NEFA from the NutriTech Study (n = 69 individuals) and the MetFlex Study (n = 40 individuals). Model simulations are shown in blue, the black crosses indicate the average of the measured data ±the standard deviation across each time point.

Firstly, to evaluate the ability of the Mixed Meal Model to capture responses to diverse meals the model was fitted to average meal challenge test data from two human intervention studies (Figure 2). The first column depicts the model fitting to the mean postprandial plasma glucose, insulin, triglyceride, and NEFA trajectories from the NutriTech Study (TNO, 2016), consisting of data from 69 overweight and obese (BMI 29.2 ± 2.8kg/m^2^) men and women (aged 50–65 years). The second column shows the model fitting to the average meal response data from the MetFlex Study (Fechner et al., 2020), an independent study containing data from 40 overweight and obese (BMI = 29.2 ± 2.7kg/m^2^) men and women (aged 51–70 years) used for validation. The model is able to capture the earlier peaks in glucose and insulin in the first 60–120 min and the more delayed postprandial increase in plasma triglyceride levels. The model also captures the postprandial dip and the subsequent overshoot in plasma NEFA that has been observed in multiple studies (Bickerton et al., 2008; Jelic et al., 2009; McQuaid et al., 2011). This NEFA overshoot and subsequent return to fasting levels can be more clearly seen in the extended model simulation displayed in Figure S1.

### Insulin sensitivity sub-groups

Secondly, to evaluate the ability of the Mixed Meal Model to infer physiologically relevant metrics of metabolic resilience from meal response data model parameters were estimated by fitting the model to average post-meal plasma glucose, insulin, triglyceride, and NEFA trajectories for insulin-sensitive and insulin-resistant sub-populations. These insulin sensitivity subgroups were defined by taking the highest and lowest tertile of individuals based on an independent measure of insulin resistance. In the MetFlex Study insulin sensitivity is measured by the M-value of the hyperinsulinemic-euglycemic clamp (DeFronzo et al., 1979), the gold standard for quantifying peripheral insulin sensitivity. In the NutriTech study, insulin sensitivity status is determined by HOMA-IR (Matthews et al., 1985), a surrogate index for whole-body insulin resistance.

**Figure 3:**
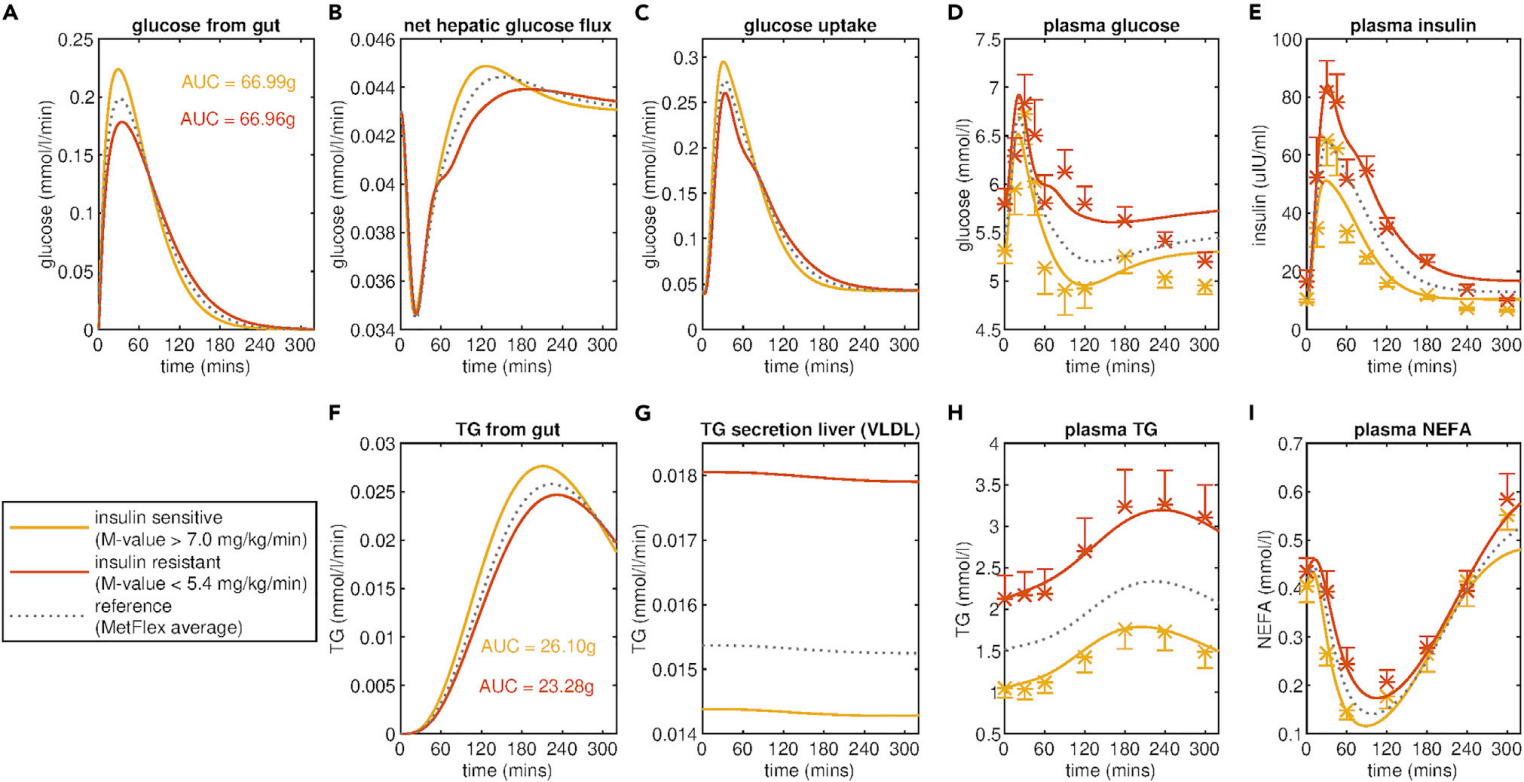
Fit of Mixed Meal Model to insulin sensitivity sub-groups in the MetFlex Study Insulin sensitivity sub-groups are defined by dividing the population of the MetFlex Study into tertiles based on the M-value of the hyperinsulinemic-euglycemic clamp. The model fitting and mean meal responses of plasma D) glucose, E) insulin, H) triglyceride, and I) NEFA for the insulin-sensitive sub-group (13 individuals with highest M-value) and the insulin-resistant sub-group (13 individuals with lowest M-value) are shown in yellow and red, respectively. The model fitting also allows for the inference of fluxes that are not directly measured including the rate of appearance of meal derived A) glucose and F) triglyceride in the plasma, B) the net hepatic glucose flux and C) the glucose uptake and G) the secretion of triglyceride from the liver. For reference, the model fit for the average meal response for all 40 individuals in the MetFlex study is shown in grey. Average measured plasma values ± standard error of the mean for glucose, insulin, triglyceride, and NEFA for the insulin-sensitive and insulin-resistant subgroups are indicated with yellow and red crosses, respectively.

Figure 3 depicts the model fitting for mean meal response for insulin-sensitive and insulin-resistant sub-groups from the MetFlex Study. Comparing the measured meal responses, it is evident that the insulin-resistant sub-group not only has higher fasting plasma glucose, insulin, and triglyceride concentrations but also higher and more prolonged postprandial responses, taking longer to return to fasting levels (Figure 3 D, E, H red). The Mixed Meal Model was capable of capturing these diverse responses to the standardized meal used in the MetFlex Study. Moreover, the Mixed Meal Model could be used to infer the rates of internal fluxes which were not directly measured. For example, the Mixed Meal Model predicts a very evident increase in the rate of triglyceride secretion from the liver (Figure 3G), with k _16_, the coefficient for triglyceride secretion from the liver, increasing 0.014 mmol/L/min in the insulin-sensitive group to 0.018 mmol/L/min for the insulin-resistant group (Table 1). Figure 3 panel C depicts the predicted glucose uptake into the peripheral tissues, indicating a dampening of the glucose uptake in the insulin-resistant group when compared to the insulin-sensitive group. This is reflected in the estimated values for k_5_, the parameter governing insulin-dependent glucose uptake into the tissues, with k_5_ decreasing from 0.142 min^−1^ for the insulin-sensitive group to 0.073 min^−1^ for the insulin-resistant group (Table 1). In addition, the estimated parameter values also indicate an increase in the rate of insulin secretion, with k_6,_ the coefficient for insulin secretion and a marker of beta-cell functionality, increasing from 2.204 min^−1^ for the insulin-sensitive group to 4.304 min^−1^ for the insulin-resistant group. An increase in beta-cell mass, and thereby insulin secretion, has been reported in individuals with insulin resistance (Chen et al., 2017). In this way the Mixed Meal Model presents a new and objective definition of metabolic resilience, reducing the multi-variate time series data collected during a meal challenge test to a three-dimensional metabolic fitness space quantifying insulin resistance, beta-cell functionality, and hepatic lipid accumulation.

**Table 1:** Estimated Mixed Meal Model parameters for insulin resistance subgroups in the NutriTech and MetFlex studies

Parameters		MetFlex			NutriTech		
		Insulin sensitive	Insulin resistant	Population average	Insulin sensitive	Insulin resistant	Population average
Number of individuals		n = 13	n = 13	n = 40	n = 22	n = 22	n = 69
$k_{5}$	Glucose uptake into tissues	0.142	0.073	0.102	0.073	0.025	0.042
$k_{6}$	Insulin secretion	2.204	4.304	2.852	1.966	2.592	2.413
$k_{11}$	Rate of lipolysis circulating TG	1.4 × 10^−4^	5.2 × 10^−5^	8.3 × 10^−5^	8.2 × 10^−5^	2.3 × 10^−5^	4.6 × 10^−5^
$K_{ATL}$	Rate of lipolysis of stored TG	0.130	0.124	0.126	0.099	0.026	0.041
$k_{16}$	TG secretion from liver (VLDL)	0.014	0.018	0.015	0.010	0.013	0.012
Mean M-value (mg/kg/min)		8.8	4.0	6.3	–	–	–
Mean HOMA-IR		2.5	4.4	3.2	2.4	6.3	4.2
Mean Hepatic lipid water ratio		–	–	–	1.4	10.3	4.5

Values for selected parameters estimated by fitting the Mixed Meal Model to meal response data for insulin-sensitivity sub-groups and the population average for the MetFlex and NutriTech studies. Insulin sensitivity subgroups are generated by taking the highest and lowest tertiles defined by the hyperinuslinemic -euglycemic clamp or the HOMA-IR index for the MetFlex and NutriTech studies, respectively. For reference sub-population averages of insulin sensitivity measured via hyperinsulinemic-euglycemic clamp and HOMA-IR index and hepatic organ fat ratio are supplied where available. A complete set of estimated parameter values can be found in Table S1.

**Figure 4:**
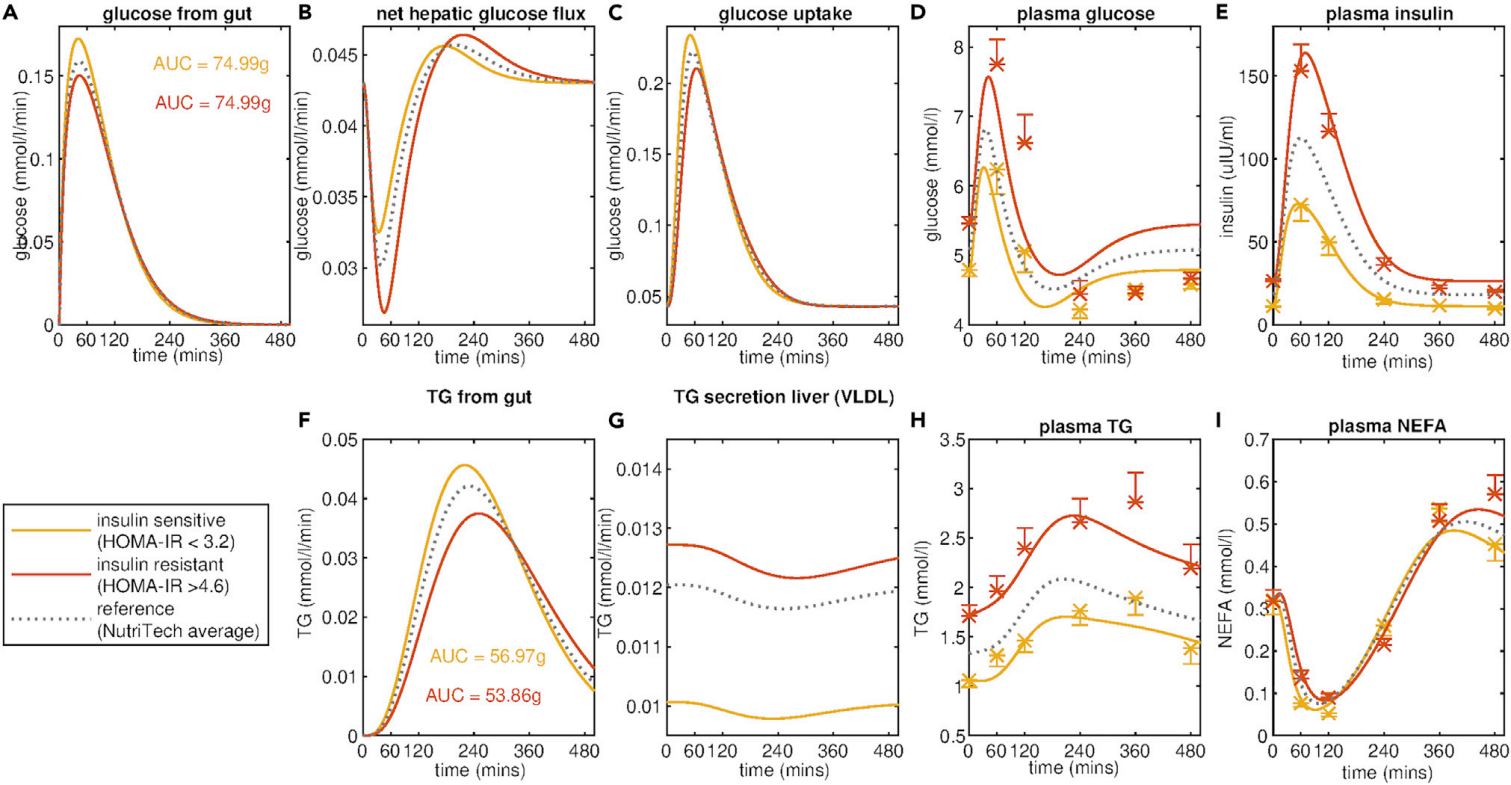
Fit of Mixed Meal Model to insulin sensitivity sub-groups in the NutriTech Study Insulin sensitivity sub-groups are defined by separating the 69 individuals from the NutriTech Study population into tertiles based on HOMA-IR value; calculated using fasting glucose and insulin measurements. The model fitting and mean meal responses of plasma D) glucose, E) insulin, H) triglyceride, and I) NEFA for the insulin-sensitive sub-group (22 individuals with lowest HOMA-IR value) and the insulin-resistant sub-group (22 individuals with highest HOMA-IR) are shown in yellow and red, respectively. The model fitting also allows for the inference of fluxes that are not directly measured including the rate of appearance of meal derived A) glucose and F) triglyceride in the plasma, B) the net hepatic glucose flux and C) the glucose uptake and G) the secretion of triglyceride from the liver. For reference the model fit for the average meal response for all 69 individuals in the NutriTech study are shown in grey. Average measured plasma values ± standard error of the mean for glucose, insulin, triglyceride and NEFA for the insulin-sensitive and insulin-resistant subgroups are indicated with yellow and red crosses, respectively.

To test the generalizability of the Mixed Meal Model as a definition and metric of metabolic resilience, this analysis was repeated for the NutriTech Study defining insulin-sensitive and insulin-resistant sub-populations using the HOMA-IR index (Figure 4). As seen for the MetFlex Study, the Mixed Meal Model predicts there is an increase in the rate of VLDL secretion from the liver, with k_16_ increasing from 0.010 mmol/L/min for the insulin-sensitive group to 0.013 mmol/L/min for the insulin-resistant group resulting in the observed increase in circulating triglyceride concentration in the insulin-resistant state. Moreover, the estimated parameter values again indicate an increase in insulin secretion (k_6_) coupled with a decrease in the rate of insulin-dependent glucose uptake into the peripheral tissues (k_5_) in the insulin-resistant group (Table 1).

### Liver fat sub-groups

**Figure 5:**
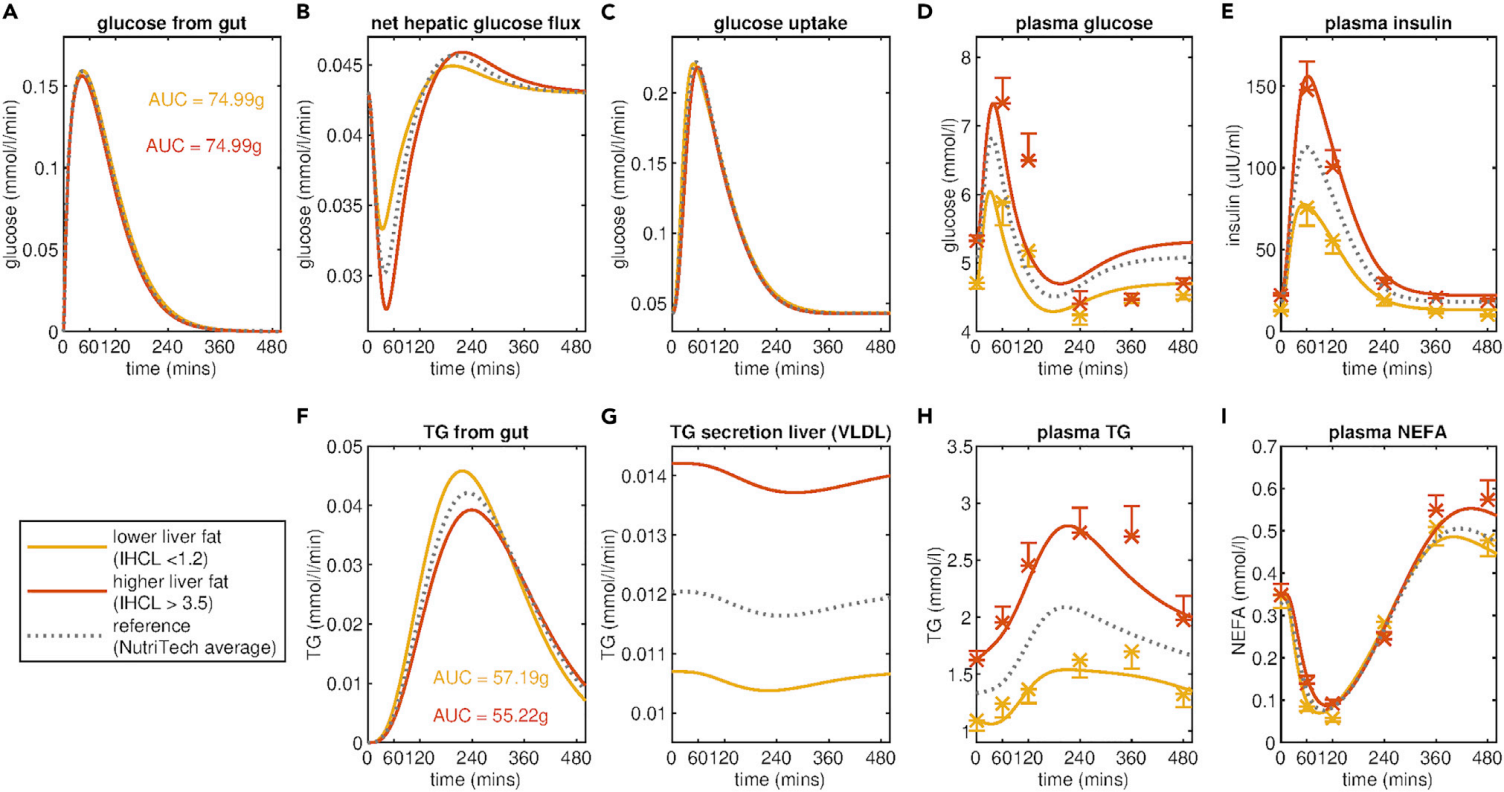
Fit of Mixed Meal Model sub-groups defined by the liver fat ratio in the NutriTech Study Liver fat sub-groups are defined by splitting the population of the NutriTech Study into tertiles based on intrahepatocellular lipid (IHCL) content. This is a measure of the ratio of the lipid to the water content of the liver measured with MRS. The model fitting and mean meal responses of plasma D) glucose, E) insulin, H) triglyceride, and I) NEFA for the lower liver fat sub-group (21 individuals with lowest IHCL) and the higher liver fat sub-group (21 individuals with highest IHCL) are shown in yellow and red, respectively. The model fitting also allows for the inference of fluxes that are not directly measured including the rate of appearance of meal derived A) glucose and F) triglyceride in the plasma, B) the net hepatic glucose flux and C) the glucose uptake and G) the secretion of triglyceride from the liver. For reference, the model fit the average meal response for all 69 individuals in the NutriTech study are shown in grey. Average measured plasma values of glucose, insulin, triglyceride, and NEFA ± standard error of the mean for the lower liver fat and higher liver fat groups are indicated with yellow and red crosses, respectively.

To explore the ability of the Mixed Meal Model to capture diverse metabolically relevant phenotypes it was also fit to the mean meal responses of individuals from the NutriTech Study stratified by higher and lower intrahepatocellular lipid content defined as the ratio of lipid to water content in the liver as quantified with proton magnetic resonance spectroscopy (Thomas et al., 2012). The results are visualized in Figure 5. The trends in the estimated parameter values between the lower liver fat group versus the higher liver fat group are in the same direction as the differences observed in the insulin-sensitive versus insulin-resistant subgroups (Table 2), with the estimated value for k_16_, the rate of endogenous triglyceride secretion from the liver being higher for the higher liver fat group than the lower liver fat group. The model-predicted increase in hepatic triglyceride secretion is evident when comparing the curves for the higher liver fat group (red) to the average for the full NutriTech population (dashed grey line) in Figure 5 panel G. Moreover, the model inferred rate of hepatic triglyceride secretion is higher for the higher liver fat group (k_16_ = 0.014 mmol/L/min) than was observed for the insulin-resistant sub-group (k_16_ = 0.013 mmol/L/min). In addition, the Mixed Meal Model derived measure of insulin sensitivity (k_5_) is lower for the higher liver fat group than the lower liver fat group.

**Table 2:** Estimated Mixed Meal Model parameters for liver fat subgroups in the NutriTech study

Parameters		NutriTech		
		Lower liver fat	Higher liver fat	Population average
Number of individuals		n = 21	n = 21	n = 69
$k_{5}$	Glucose uptake into tissues	0.067	0.028	0.042
$k_{6}$	Insulin secretion	2.351	2.961	2.413
$k_{11}$	Rate of lipolysis circulating TG	8.0 × 10^−5^	2.9 × 10^−5^	4.6 × 10^−5^
$K_{ATL}$	Rate of lipolysis of stored TG	0.057	0.038	0.041
$k_{16}$	TG secretion from liver (VLDL)	0.011	0.014	0.012
Mean HOMA-IR		2.8	5.3	4.2
Mean Hepatic lipid water ratio		0.7	10.9	4.5

Values for selected parameters estimated by fitting the Mixed Meal Model to meal response data for the population average and intrahepatocellular lipid sub-groups from the NutriTech study. Liver fat subgroups are generated by taking the highest and lowest tertiles defined by the intrahepatocellular lipid to water ratio measured using MRS. A complete set of estimated parameter values can be found in Table S1.

### Physiology-informed regularization

When estimating parameter values, the cost function has been extended to not only account for the quality of the model fit to the measured meal response data but also to ensure the parameters produce biologically plausible behavior. This is achieved by supplementing the cost function with terms to ensure all exogenous glucose and triglyceride appear within four and ten hours of meal consumption, respectively and that the system returns to the measured fasting steady state by twelve hours post-ingestion. We have termed these additional penalties physiology-informed regularization. In Figure S2 the effect of the physiology-informed regularization is visualized. In panel I, we can see that the model trained without regularization (red curve) finds a new steady state owing to the influence of the data points measured during the postprandial overshoot in NEFA between 360 and 480 min, also introducing an erroneous spike in the predicted plasma NEFA concentration in the first 60 min. By including a term in the cost function that penalizes model simulations where the model-predicted plasma NEFA concentration at 0 min differs from the measured fasting plasma NEFA concentration (blue curve) the parameter estimation algorithm is guided toward regions of the solution space where the model steady state reflects the measured fasting values.

## Discussion

The Mixed Meal Model captures deviations in the post-meal excursion of plasma glucose, insulin, and triglyceride concentrations that are indicative of features of metabolic resilience such as insulin resistance status, beta-cell functionality, and intrahepatic lipid accumulation. Moreover, trends in the resulting parameter estimates are reflective of the expected difference in the underlying physiology. The estimated values for k_5_, the parameter governing insulin-mediated uptake of glucose into the tissues, is 50% lower in the insulin resistant than in insulin-sensitive sub-populations in the MetFlex Study. The Mixed Meal Model also predicts an increase in the rate of insulin secretion, compensating for the decrease in insulin sensitivity in the tissues (Cheng et al., 2019; Tura et al., 2011). To further validate the Mixed Meal Model predictions we repeated these analyses by applying the Mixed Meal Model to meal responses for insulin sensitivity subgroups from the NutriTech study defined using the HOMA-IR index. The insulin-resistant group consists of 22 individuals with a HOMA-IR value greater than 4.6 and the insulin-sensitive grouping all have a HOMA-IR value less than 3.2. Again, we see the same trend in parameter estimates, with the model-predicted insulin sensitivity (k_5_) decreasing from 0.073 min^−1^ to 0.025 min^−1^ from the insulin-sensitive to insulin-resistant group, respectively, and the model inferred rate of insulin secretion (k_6_) increases from 1.966 min^−1^ to 2.592 min^−1^. The relationship between the parameter k_5_ and independent insulin-resistant measures such as the hyperinsulinemic-euglycaemic clamp and HOMA-IR is in-line with the findings of a recent study by Edrős et al. who report a statistically significant correlation of 0.68 between k_5_ and the Matsuda index (Matsuda and DeFronzo, 1999) when applying a concordant model of glucose and insulin dynamics to OGTT responses from a large population of overweight individuals (n = 738) (Erdős et al., 2021). Although we see consistent trends in estimated model parameters when comparing insulin-sensitive and insulin-resistant groups within the MetFlex and Nutritech studies, the numerical values of the parameter estimates do differ between the study populations. Further application of the Mixed Meal Model to data from additional studies would not only confirm reliable ranges for each parameter but potentially allow for the determination of parameter cut-offs that would be indicative of metabolic disease states as has been achieved for other surrogate measures of glycemic control such as HOMA-IR (Gayoso-Diz et al., 2013).

Terms introduced to capture changes in plasma triglyceride predict an increase in the rate of endogenous triglyceride secretion from the liver (Table 1, k_16_). Moreover, the intrahepatocellular lipid measured using MRS increases from an average of 1.4 for the insulin-sensitive group to 10.3 for the insulin-resistant group suggesting the model is capturing changes in the meal response that are indicative of increased liver fat accumulation. This increase in the rate of endogenous triglyceride secretion in insulin-resistant state has previously been observed in stable isotope tracer studies (Adiels et al., 2007). This suggests estimates of k_16_ from the Mixed Meal Model have the potential to provide a surrogate measure of intrahepatic lipid accumulation as measured with MRS, however further validation considering personalized models on independent study populations would be necessary to confirm that the observed relationship is reproducible.

To further explore the ability of the Mixed Meal Model to differentiate between metabolic resilience phenotypes, we repeated the above analysis for sub-populations of the NutriTech study defined by the hepatic organ fat ratio. The increase in the predicted rate of endogenous triglyceride secretion from the liver, our proposed surrogate measure of hepatic fat accumulation, relative to the NutriTech population average is twice as large for the higher liver fat group (Table 2, k_16_) than the insulin resistance group (Table 1, k_16_). In addition, the decrease in model-predicted insulin sensitivity relative to the average reference is less for the higher liver fat group than for the insulin-resistant group. The smaller difference in model-predicted insulin resistance status between the higher and lower liver fat sub-groups in NutriTech is reflective of range of HOMA-IR value for the groupings, with the difference between the lower and higher liver fat being 2.5 units whereas the difference between the insulin sensitivity sub-groups being 3.9 units (Table 2). These differences in parameter estimates between the higher liver fat and insulin-resistant subgroup in NutriTech indicate that the Mixed Meal Model can capture subtle, yet physiologically relevant, differences between these phenotypes in the post-meal trajectories of glucose, insulin, triglyceride, and NEFA. Moreover, just 13 individuals were common to both the insulin-resistant (n = 22) and the higher liver fat (n = 21) groups, demonstrating that the determination of a metabolic impairment is highly dependent on the metric being used, reflecting the heterogeneity we see in the manifestation of metabolic deteriorations associated with obesity. Currently in research, an array of metrics and indices that rely on glucose concentrations at a single time point or as a simple function of glucose and insulin are regularly employed to quantify glucose tolerance or insulin resistance independently. Using the Mixed Meal Model we decompose the high-dimensional meal challenge test data into a subset of parameters, simultaneously quantifying metabolic resilience according to three axes, namely insulin resistance status, beta-cell functionality, and hepatic lipid accumulation. In this way, we can differentiate between subtle differences in the meal response dynamics that are indicative of distinct metabolic sub-phenotypes.

There is growing interest surrounding the field of precision nutrition, whereby dietary interventions will be targeted toward an individual’s specific metabolic aberrations (Ben-Yacov et al., 2021; Berry et al., 2020; Gijbels et al., 2021; Zeevi et al., 2015). In order to achieve this transition toward the provision of personalized interventions it is necessary to be able to objectively quantify metabolic resilience. A recent study by Erdős et al. outlined a pipeline for the individualization of computational models such as the Mixed Meal Model (Erdős et al., 2021). In future work, the generation of personalized Mixed Meal Models could allow for the individualized assessment of metabolic resilience, quantifying three features of metabolic resilience: 1) hepatic triglyceride secretion, 2) insulin sensitivity, and 3) the rate of insulin secretion under physiologically relevant conditions. Each component has been studied independently and compared with their respective gold standard methods for clinical assessment. In this way, the M3al Model can allow for the identification of specific metabolic deteriorations and individualized assessment of intervention success, thereby supporting the transition toward precision nutrition.

In this study, the Mixed Meal Model is successfully applied to mixed meal data from two independent studies, indicating that the model is generalizable. Moreover, the macro-nutrient composition of the meals differs between the two studies, with the shake from the NutriTech Study consisting of 75g of glucose, 60g of palm oilen (lipid), and 20g of protein whereas the meal from the MetFlex Study consisting of a mixture of whole milk, fruit concentrate, egg yolk, safflower oil, and unsalted butter containing 67g of glucose, 36g of lipid, and 12g of protein. In this way, we show the Mixed Meal Model definition of metabolic resilience is robust and applicable to diverse meals. Although, both of the meals used in our analyses were liquid mixed meal shakes, with comparatively simple rates of appearance, they are representative of the types of meal challenges commonly used in a research setting (Wopereis et al., 2017). However, validation of the model on responses to meals containing food products with a more complex food matrix would be beneficial. In addition, both the NutriTech and MetFelx study populations consist of overweight and obese men and women (ages 50 to 71 years and a BMI of 24.9 to 35.8 kg/m^2^) with no overt clinical metabolic disease. Further validation of the Mixed Meal Model on more diverse populations such as lean healthy individuals or patients with type 2 diabetes mellitus is necessary to confirm both the robustness of the model fit and range over which the estimated parameter values maintain physiologically relevance. In this study we have successfully applied the model to a range of meal responses indicative of insulin-sensitive and resistant states as well as lower and higher liver fat accumulation, consequently we expect that the Mixed Meal Model is sufficiently flexible to be directly applied to these different study populations. In some instances, for example, if fitting to individuals with type 1 diabetes mellitus, it may be necessary to adjust the values of fixed parameter or alter which parameters are estimated to better represent the underlying physiology. In these instances, we would recommend repeating the local parameter sensitivity analysis and identifiability analyses outlined in the STAR Methods section to ensure the model remains parsimonious.

Triglycerides are transported in the blood in the form of lipoprotein particles such as chylomicrons or very low-density lipoproteins, each with their own distinct properties and kinetics (Adiels et al., 2007; Bickerton et al., 2007; Packard et al., 2000, 2020). However, measuring the triglyceride flux across individual lipoprotein classes can be complex and time-consuming, often necessitating the use of expensive tracer measurements. As a result, these measurements are rarely generated for standard meal challenge tests. To make the Mixed Meal Model as generalizable as possible we have elected to group endogenous and dietary derived triglyceride into a single generic triglyceride pool. In this way, the model can be parameterized using measurements of plasma triglyceride alone. In future work, it would be possible to decouple the endogenous and dietary-derived triglyceride terms to account for specific lipoprotein kinetics. In this study we find consistent trends between the estimated values for parameters governing the rates of endogenous triglyceride secretion from the liver (k_16_) or LPL lipolysis of circulating triglyceride (k_11_) and independent measures of health such as insulin resistance and liver fat accumulation; however, we have not been able to quantitatively validate the predicted model fluxes. Although the term used to describe the rate of LPL lipolysis has previously been validated using arterio-venous measurements coupled with a palmitate tracer the equation describing endogenous triglyceride secretion from the liver is new. Should such data become available, the application of the Mixed Meal Model to challenge test data which makes use of multiple stable isotope tracer protocols to label both endogenous triglyceride secretion and the rate of LPL lipolysis could allow for the validation of these predicted fluxes.

When estimating parameters for the Mixed Meal Model from measured meal response data we employed physiology-informed regularization, whereby the cost function used to fit the model to the data was extended to penalize unfavorable behavior, such as nutrient dumping or a failure to return to the measured steady state. The inclusion physiology-informed regularization has a minimal impact on the fit of the model to the measured meal response data (Figure S2). However, the true benefit of this physiology-informed regularization becomes more evident when looking at the longer-term dynamics (9-15 hours) of triglyceride and NEFA in the Mixed Meal Model simulation; the model trained without regularization finds a new erroneous steady state (Figure S2, red) whereas the model trained with regularisation displays the characteristic postprandial NEFA overshoot reported in the literature (Jelic et al., 2009) and then returns to steady-state concentrations. We purport that physiology-informed regularization can be a particularly powerful tool when estimating model parameters, particularly in instances with sparse sampling frequency.

The Mixed Meal Model expands on classical models of glucose and insulin dynamics by explicitly accounting for interactions with lipid species, allowing for the detection of early changes in postprandial triglyceride dynamics that may be predive for future disease risk. However, the model still does not describe the effects of protein ingestion, dietary fibers, and other hormones such as glucagon and GLP1 on glucose and lipid metabolism. Studies have demonstrated the modulatory effect of dietary derived protein on both insulin secretion and endogenous glucose production (Sloun et al., 2020). Moreover, the impact of incretin hormones such as GLP1 and GIP on both insulin secretion and satiety has been widely reported (Baggio and Drucker, 2007), with GLP1-agonists now being regularly employed in the treatment of type 2 diabetes mellitus (Man et al., 2009). In future work, the integration of protein kinetics, as well as terms to explicitly model the effects of incretin hormones in the Mixed Meal Model, may further improve the ability of the model to capture physiologically relevant features of metabolic health from meal response data.

In summary, in this study, we introduce a computational model capable of quantifying features of metabolic resilience from meal challenge test data. As the Mixed Meal Model introduces terms explicitly accounting for the role of triglyceride and NEFA in the glucose and insulin system it provides a more holistic view of metabolic resilience than existing summary measures, models, and indices. Application of our model to meal challenge test data from two independent human studies indicates that not only is the Mixed Meal Model generalizable to meals with different macro-nutrient compositions but that the estimated parameter values capture distinct features of metabolic health such as endogenous triglyceride secretion, insulin sensitivity, and beta-cell functionality. In this way, our Mixed Meal Model provides a new and objective definition of metabolic resilience.

### Limitations of the study

While we have shown consistent trends between estimated parameter values and independent measures of metabolic health for sub-populations defined by insulin resistance status and liver fat accumulation we have not quantitatively validated the predicted model fluxes. In future work, the use of challenge test data incorporating a stable isotope tracer protocol labeling glycerol or palmitate would allow for the validation of these model fluxes, particularly for newly introduced terms such as the rate of endogenous triglyceride secretion. In this study we have successfully applied the Mixed Meal Model to liquid meals with different macro-nutrient compositions, further validation of the Mixed Meal Model on responses to meals with complex food matrices would be necessary to apply the model to free-living conditions. We present a model accounting for the insulin-mediated interactions between glucose, triglyceride, and NEFA; however, the Mixed Meal Model fails to account for the role of dietary protein or incretin hormones such as GIP and GLP1 which have been shown to impact the postprandial insulin response.

## STAR★Methods

### Key resources table

**Table:** 

REAGENT or RESOURCE	SOURCE	IDENTIFIER
**Software and algorithms**		

MATLAB 2019b	The MathWorks Inc.	https://nl.mathworks.com/products/matlab.html

**Other**		

Original Mixed Meal Model code	This paper	https://github.com/shauna-odonovan/Mixed_Meal_Model

### Resource availability

#### Lead contact

Further information and requests for resources should be directed to and will be fulfilled by the lead contact Shauna D. O’Donovan (s.d.odonovan@tue.nl)

#### Material availability

No new materials or reagents were generated during this study.

### Method details

#### Data

##### NutriTech

The Nutritech Study, funded as part of the European Union 7th Framework Programme (www.clincaltrials.gov record no. NCT01684917), aimed to better phenotype human volunteers in response to standardised challenge tests in dietary intervention studies (TNO, 2016). As part of this study 72 overweight and obese (mean BMI 29.7 ± 2.7 kg/m^2^) men and women (48.6% male) with an average age of 59.2 ± 4.2 years were recruited. Prior to the intervention period study participants underwent a high-fat, high-glucose liquid meal challenge test (75g glucose, 60g lipid (palm olein), 20g Protifar as milk protein concentrate (Nutricia, Utrect, the Netherlands) (Wopereis et al., 2017)). The meal challenge test began at 9AM following a 12-h over-night fast. The liquid meal was ingested within 5 min and blood was collected at 0, 60, 120, 240, 360, and 480 min in which glucose, insulin, triglyceride, and non-esterified free fatty acids (NEFA) concentrations were measured. In addition, participant’s body composition, including liver fat content, were assessed with MRI and spectroscopy on a 1.5 T multinuclear system (Philips, Eindhoven, the Netherlands) as previously described (Thomas et al., 2012). All subjects gave written informed consent before participating in the NutriTech Study. The NutriTech Study was performed in accordance with the Declaration of Helsinki and received ethical approval from the Brent Ethics Committee (REC ref:12/LO/0139).

##### MetFlex

MetFlex is dietary intervention study in which 40 middle-aged (50–70 years) healthy, but overweight or obese (BMI 25–35 kg/m^2^) men and women (47.5% male) were randomly assigned to either a western diet or healthy diet (low in high-glycaemic carbohydrates and rich in fruits, vegetables, fibres, and polyunsaturated fats) for six weeks (Fechner et al., 2020). Prior to the diet intervention period all study participants underwent a liquid mixed meal challenge test (67g glucose, 36g lipid,12g protein, as described in Table S1 of Fechner et al., 2020) following an overnight fast. Blood samples were collected at 0, 15, 30, 45, 60, 90, 120, 180, 240, and 300 min following consumption of the liquid meal shake. Plasma glucose and insulin concentrations were determined at all time points. Plasma triglyceride and NEFA concentrations were measured at 0, 30, 60, 120, 180, 240, and 300 min. In addition, all study participants underwent a 2.5 h 1-step hyperinsulinemic-euglycemic clamp with an insulin infusion rate of 40mU/m^2^/min to quantify peripheral insulin sensitivity. All participants gave their written and informed consent prior to the start of the study. The study was conducted according to the guidelines stated in the Declaration of the Helsinki and the protocol was approved by the medical ethical committee of Maastricht University Medical Centre+ (MUMC+) and registered at www.clincaltrials.gov as NCT02519127.

#### Model formulation

##### Glucose and insulin kinetics

Several well validated glucose-insulin models are currently available in the literature; from simple minimal models that describe changes in plasma glucose at a whole-body level (Bergman et al., 1979; Eichenlaub et al., 2019) to more detailed multi-compartmental models describing glucose metabolism across multiple tissues (Dalla Man et al., 2007; Pearson et al., 2016; Sips et al., 2015). One such model, the Eindhoven Diabetes Education Simulator (E-DES) is a comparatively simple three compartment physiology based mathematical model describing postprandial glucose and insulin dynamics in the gut, plasma, and interstitial fluid (Maas et al., 2015). The E-DES model has previously been applied to describe postprandial plasma glucose and insulin excursions in response to a diverse range of complex meals (Rozendaal et al., 2018) and more recently has been shown to capture the considerable inter-individual heterogeneity in oral glucose tolerance test responses (Erdős et al., 2021). In the E-DES model glucose appears in the plasma by either endogenous glucose released from the liver or as exogenous glucose from a meal via the gut (Figure 1). Uptake of plasma glucose into tissues such as the skeletal muscle, adipose tissue, and brain occurs at an insulin dependent and independent rate and are described as a collective glucose sink. Insulin secretion in response to changing plasma glucose concentrations is described using a proportional-integral-derivative controller. A more detailed model formulation can be found in Section S1.

##### Triglyceride kinetics

Plasma triglycerides have two primary sources; exogenous triglyceride from dietary intake transition through the gut and lymphatic system (described as three transition compartments) before appearing in the plasma as chylomicron triglyceride ($TG_{gut})$. Endogenous triglyceride is secreted from the liver as very low-density lipoprotein (VLDL) particles ($TG_{liver})$ which is inhibited by insulin in lean, healthy individuals (McQuaid et al., 2011). The effect of insulin on VLDL secretion has been shown to be attenuated in insulin resistance and with increased hepatic fat (Adiels et al., 2007, 2008; Avramoglu et al., 2006) content. Both chylomicron and VLDL triglycerides are removed from the plasma through hydrolysis by lipoprotein lipase (LPL) which is stimulated by insulin ($TG_{LPL})$ and the resulting NEFA is taken up into tissues including the skeletal muscle and adipose tissue (O’Donovan et al., 2019). To make our meal model as generalisable as possible we have elected to combine both chylomicron and VLDL into a single generic triglyceride pool, in this way the model can be fit to postprandial plasma triglyceride measurements without the need to quantify specific lipoprotein subfractions. Consequently, the rate of change of plasma triglyceride concentration ($TG_{\mathrm{PL}})$ is described as such;

$$\frac{dTG_{\mathrm{PL}}}{dt}\;=\;TG_{gut\;}\;+\;TG_{liver}\;-\;TG_{LPL}$$

Where,

$$TG_{liver\;}=\;k_{16}\;-\;k_{15}\cdot \left(I_{d4}-\;I_{b}\right)$$

Here, $k_{16}$ is the basal rate of secretion of endogenous triglyceride from the liver and $k_{15}$ governs the effect of insulin on VLDL secretion.$\;I_{d4}$ is the delayed insulin signal that inhibits triglyceride secretion and $I_{b}\;$ is the basal (fasting) insulin concentration. $TG_{LPL}$, the term describing LPL lipolysis of circulating triglyceride has previously been validated using arterio-venous flux measurements (O’Donovan et al., 2019).

##### NEFA kinetics

In the fasting state NEFA enters the plasma from the adipose tissue ($NEFA_{ATL})$. The release of NEFA from triglyceride stores in the adipose tissue is inhibited by insulin in the postprandial state. In addition, a small proportion ($f_{spill})$ of NEFA released by LPL lipolysis of circulating triglyceride spills-over into the plasma. Model terms describing the fractional spill-over and release of NEFA from the adipose tissue have previously been validated (O’Donovan et al., 2019) using arterio-venous palmitate tracer measurements collected across the abdominal subcutaneous adipose tissue. NEFA is taken up into tissues at a rate ($k_{12})\;$ proportional to the plasma NEFA concentration (Sips et al., 2015). The rate of change of plasma NEFA concentrations is described in the following way;

$$\frac{d\mathrm{NEF}A_{\mathrm{PL}}}{dt}\;=\;NEFA_{ATL\;}\;+\;3\;f_{spill}\cdot TG_{LPL}\;-\;k_{12}\cdot \mathrm{NEF}A_{\mathrm{PL}}$$

Where $\mathrm{NEF}A_{\mathrm{PL}}$ is the plasma NEFA concentration and $TG_{LPL}$ is the rate of LPL lipolysis of circulating triglyceride described above.

A complete list of model equations can be found in Section S1.

#### Parameter estimation

The final mixed meal model consists of 13 ordinary differential equations with 25 model parameters. To achieve numerically reliable parameter estimates from data it is necessary to minimise the complexity of the model being applied. Local parameter sensitivity analyses, whereby each parameter was varied 50% in both directions from the average estimate, was conducted to determine which parameters have substantial effect on the model output (Figures S4–S26). Non-sensitive parameters were fixed to values reported in their respective publications (O’Donovan et al., 2019; Rozendaal et al., 2018). Basal glucose and insulin values were fixed to the measured plasma glucose and insulin values following an overnight fast, as advised for the E-DES model (Maas et al., 2015). This analysis resulted in a model with nine parameters that will be estimated from the data. Subsequent profile likelihood analysis (Raue et al., 2009) showed that these nine parameters were identifiable given the six time-point meal challenge test data in the NutriTech Study (Figure S3). A full set of model parameters can be found in Table S2.

Parameter values were estimated from data by minimizing the below combined cost function C(θ) using *lsqnonlin*, (MATLAB, 2019b, The MathWorks Inc., Natick, Massachusetts, United States) a local, gradient-based least square solver. To avoid becoming trapped in erroneous local minima, the optimal parameter sets were obtained following twenty-five initializations of the optimization algorithm using Latin hypercube sampling of the solution space.

$$C\left(\theta \right)=\;\overset{M}{\underset{i=1}{\sum }}\overset{T_{i}}{\underset{j=1}{\sum }}{\left(\frac{y_{i,j}\left(\theta \right)-\;y_{i,j}^{obs}}{\max\left(y_{i}^{obs}\right)}\right)}^{2}\;$$

Where M is the number of measured metabolites, in this case glucose, insulin, triglyceride, and NEFA. $T_{i}\;$ is the number of time points for which measured data is available for metabolite i. $y_{i,j}\left(\theta \right)$ denotes the model prediction for metabolite i at time point j for a given parameter vector θ and $y_{i,j}^{obs}$ denotes the corresponding measured concentration of metabolite i at time point j. To account for the difference in scales between the metabolites the difference between the model simulation and the observed measurements for the error function are weighted by the maximum observed value for the given metabolite.

#### Physiology-informed regularisation

Regularisation is the process by which additional information about a system is supplied during the parameter estimation procedure in-order to solve an ill-proposed problem or to prevent overfitting. In this study, a number of additional terms are added to the cost function C(θ) to penalise undesirable behaviours, such as nutrient dumping or a failure of the model simulation to return to steady state, thereby guiding the parameter estimation algorithm towards regions of the solution space which produce physiologically plausible behaviour. We have dubbed this form of regularisation as physiology-informed regularisation. To ensure that the full mass of glucose and triglyceride administered in the meal ($G_{meal}$ and $TG_{meal}$ respectively) appear in the plasma and are not dumped from the gut compartment of the model two additional constraints $AUC_{G}$ and $AUC_{TG}$ are placed on the model fitting. Within these constrains the area under the curve of glucose appearance from the gut within the first four hours (Anderwald et al., 2011) after the meal consumption should equal the glucose content of the meal and the complete triglyceride appearance via the lymphatic system should occur within 10 h (Bickerton et al., 2007; Ruge et al., 2009). Two additional constraints are included to ensure triglyceride and NEFA return to steady state or fasting values. Hence, the modified cost function ($C^{*}\left(\theta \right))$ for parameter estimation becomes;

$$C^{*}\left(\theta \right)=\;C\left(\theta \right)+\left(AUC_{G}{|}_{240}-G_{meal}\right)+\;\left(AUC_{TG}{|}_{600}-\;TG_{meal}\right)\;+\left(TG_{PL}{|}_{720}-TG_{b}\right)\;+\left(NEFA_{PL}{|}_{0}-NEFA_{b}\right)\;$$

Where $TG_{b}$ and $NEFA_{b}$ are the measured fasting plasma concentrations of triglyceride and NEFA respectively.

## Data Availability

• The NutriTech and MetFlex meal challenge test data used in this study are unsuitable for public deposition due to ethical restrictions and privacy of participant data. The MetFlex data is available from Unilever for any interested researcher who meets the criteria for access to confidential data please contact Doris M. Jacobs (Doris.Jacobs@unilever.com) or Anne J. Wanders (Anne.Wanders@unilever.com). NutriTech data is available to qualified researchers via the Nutritional Phenotypes Database (https://studies.dbnp.org/NutriTechHIS) or by contacting Lydia A. Afman (lydia.afman@wur.nl). • All original code is provided in the supplementary materials (Data S1) and has also been deposited in GitHub (https://github.com/shauna-odonovan/Mixed_Meal_Model). • Any additional information required to re-analyse the data reported in this paper is available from the lead contact upon request.

## Supplemental information

Document S1. Figures S1–S26 and Tables S1 and S2 and Section S1 and S2

Data S1. Mixed meal model code
