# Supplementary material for: Quantifying the contribution of triglycerides to metabolic resilience through the mixed meal model
Source: iScience. 2022 Oct 3;25(11):105206. doi: 10.1016/j.isci.2022.105206 (PMC9587016; doi:10.1016/j.isci.2022.105206)
Supplement: Document S1. Figures S1–S26 and Tables S1 and S2 and Section S1 and S2 [file mmc1.pdf]

iScience, Volume *25*

Supplemental information

Quantifying the contribution of triglycerides

to metabolic resilience through the mixed meal model

Shauna D. O'Donovan, Balázs Erdős, Doris M. Jacobs, Anne J. Wanders, E. Louise Thomas, Jimmy D. Bell, Milena Rundle, Gary Frost, Ilja C.W. Arts, Lydia A. Afman, and Natal A.W. van Riel

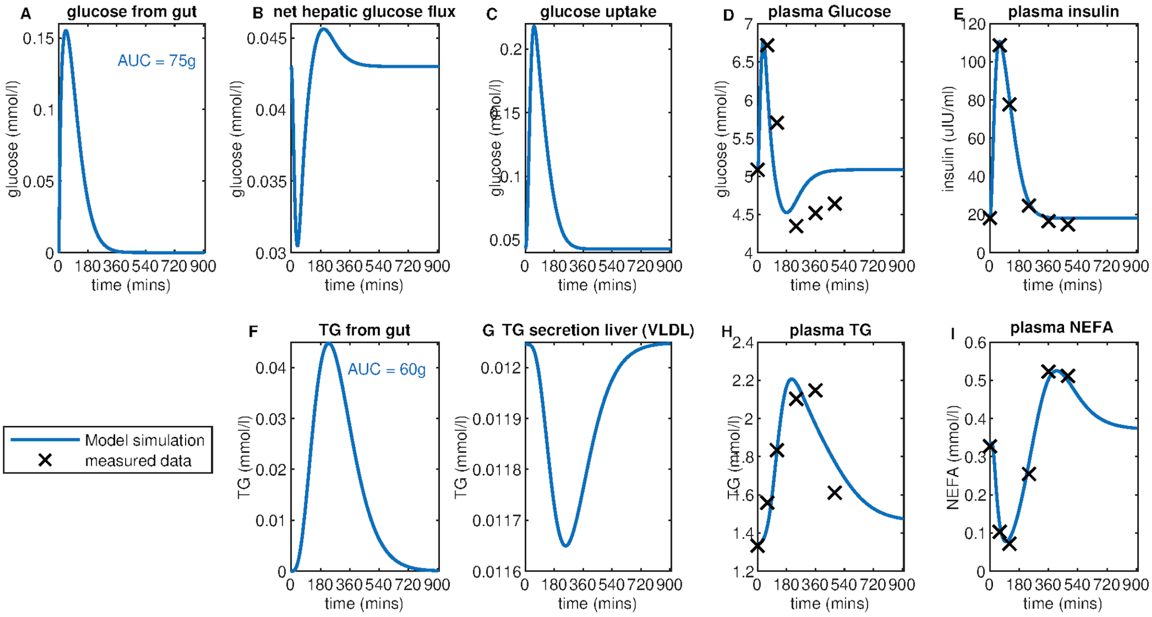

Supplementary Figure S1 : Extended simulation of Mixed Meal Model presented in Figure 2

Extended visualisation of the postprandial response of the Mixed Meal Model for 15 hours following consumption of a liquid mixed meal (blue). Model parameters for this simulation are estimated by fitting the model to average meal response of plasma glucose, insulin, triglyceride, and NEFA from the NutriTech Study (n=69 individuals) shown with the black crosses.

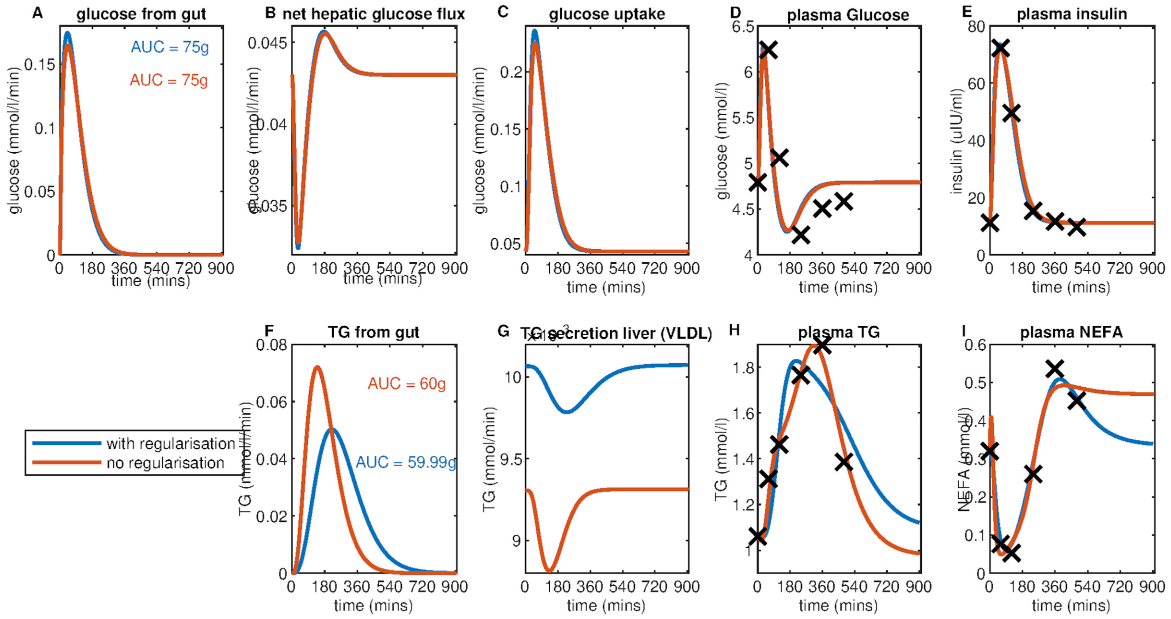

Supplementary Figure S2: Visualisation of Mixed Meal Model fit to average meal response of the NutriTech study with and without use of physiology-informed regularisation described in STAR Methods.

Mixed Meal model trained with physiology-informed regularisation is shown in blue, the red curve depicts the Mixed Meal Model fit to the average meal response data from the NutriTech Study without physiology-informed regularisation.

2

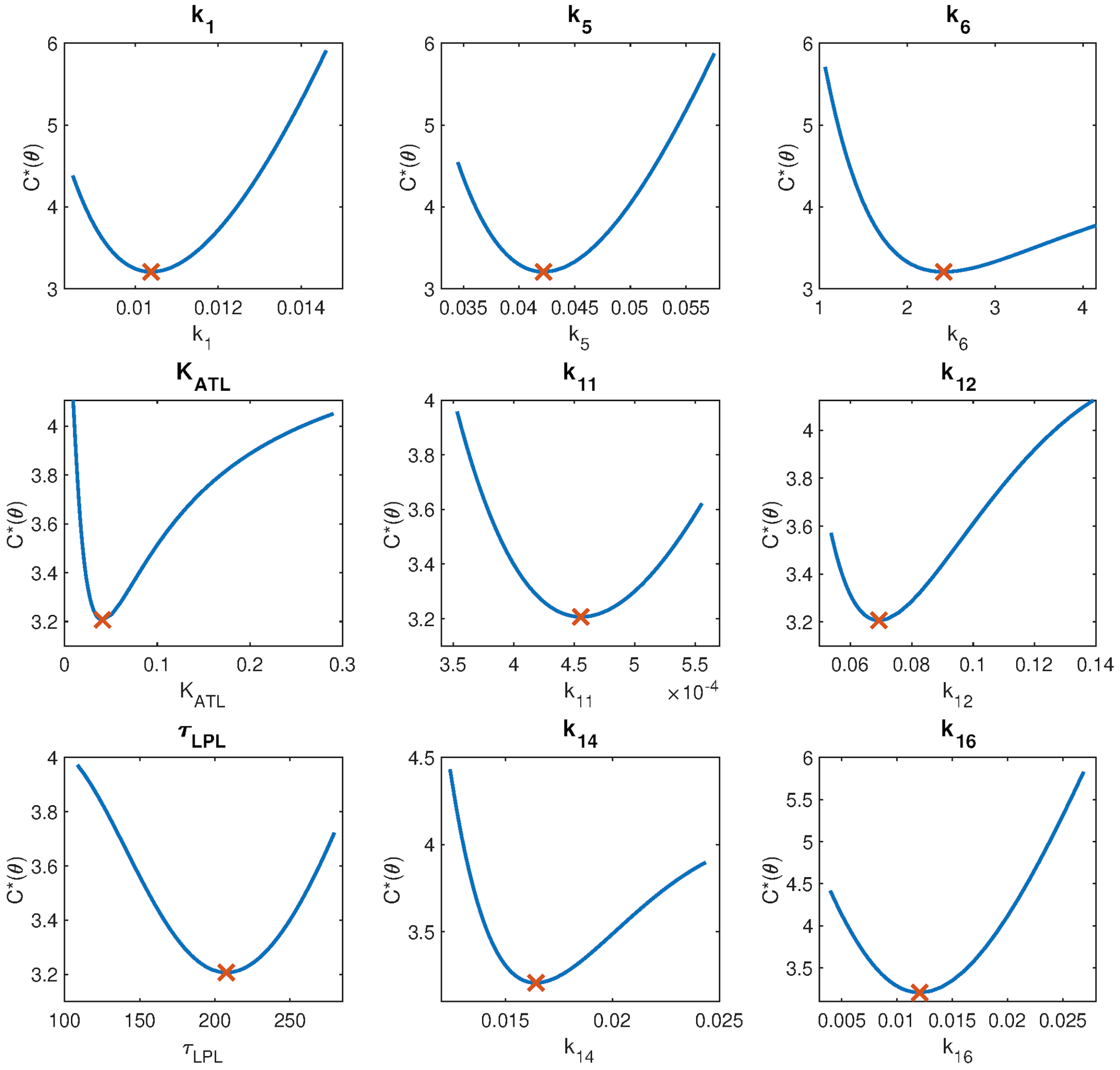

Supplementary Figure S3 : Results of profile likelihood analysis for parameters estimates of Mixed Meal Model described in STAR Methods.

The estimated parameter values for the average meal response in the NutriTech study are indicated with a red cross, the value of the cost function $C*(\theta)$ resulting from iteratively adjusting each parameter value in turn are indicated with the blue curves. An increase in the $C*(\theta)$ profile as you move away from the optimal parameter value indicates a local minimum has been reached in the parameter estimation procedure. Here all 9 of the estimated model parameters appear to be identifiable given the sampling schedule of the NutriTech mixed meal.

Supplemental Table S1: Complete set of estimated Mixed Meal Model parameters for NutriTech and MetFlex studies shown in Tables 1 and 2

parameters		MetFelx - insulin sensitivity			NutriTech - insulin sesntivity			NutriTech - liver fat		
		Insulin sensitive (n=13)	Average (n=40)	Insulin resistant (n=13)	Insulin sensitive (n=22)	Average (n=69)	Insulin resistant (n=22)	Low liver fat (n=21)	Average (n=69)	High high fat (n=21)
k_1	Rate of appearance of glucose from meal.	0.0164	0.0147	0.0113	0.0109	0.0104	0.0101	0.0101	0.0104	0.0106
k_5	Glucose uptake into tissues.	0.1421	0.1017	0.0726	0.0728	0.0422	0.0253	0.0666	0.0422	0.0277
k_6	Insulin secretion.	2.2040	2.8524	4.3039	1.9656	2.4134	2.5917	2.351	2.4134	2.9609
K_{ATL}	Rate of lipolysis of stored TG.	0.1300	0.1262	0.1241	0.0991	0.0411	0.0255	0.0567	0.0411	0.0378
k_{12}	NEFA uptake into tissues.	0.075	0.0598	0.0510	0.0749	0.0693	0.0597	0.0793	0.0693	0.0556
τ_{LPL}	Lipid insulin delay	288.606	319.894	348.278	186.336	207.618	236.848	168.976	207.617	248.845
k_{14}	Rate of appearance of TG from meal.	0.0189	0.0172	0.0162	0.0177	0.0164	0.0144	0.0181	0.0164	0.0155
k_{16}	TG secretion from liver (VLDL).	0.0144	0.0153	0.0180	0.0101	0.0120	0.0127	0.0107	0.0120	0.0142
k_{11}	Rate of lipolysis of circulating TG.	0.0014	0.0008	0.0005	0.0008	0.0005	0.0002	0.0008	0.0005	0.0003
Age (years)		61.2±7.1 (51,70)	61.4±6.3 (51,70)	62.8±5.9 (52,69)	58.4±5.2 (50,65)	59.2±4.2 (50,65)	59.5±3.3 (51,65)	59.1±4.5 (52,64)	59.2±5.2 (50,65)	59.2±3.4 (51,64)
BMI (kg/m^2)		28.6±3.2 (25.3,34.2)	29.2±2.7 (24.9,34.1)	30.6±2.4 (24.9,33.9)	27.7±2.2 (24.9,34.0)	29.2±2.8 (29.4,35.8)	29.9±3.3 (25.2,35.8)	28.1±2.5 (24.9,34.6)	29.2±2.8 (24.9,35.8)	29.8±3.0 (25.6,35.8)
sex (% male)		38.5	47.5	46.2	59.1	58.0	77.3	47.6	58.0	52.4
HOMA-IR		2.5±1.0 (1.3,4.6)	3.2±2.5 (1.1,17.4)	4.4±4.0 (1.7,17.4)	2.4± 0.6 (0.7,3.1)	4.2±1.8 (0.7,9.0)	6.3±1.3 (4.8,9.0)	2.8±1.1 (0.7,5.9)	4.2±1.8 (0.7,9.0)	5.3±1.7 (2.8,8.6)
M-value (mg/kg/min)		8.8±1.6 (7.1,12.2)	6.3±2.3 (2.4,12.2)	4.0±0.9 (2.4,5.4)	-	-	-	-	-	-
Liver fat (IHCL)		- -	- -	- -	1.4±1.7 (0.3,6.7)	4.5±6.8 (0.3,37.6)	10.3±9.8 (0.9,37.6)	0.7±0.3 (0.3,1.1)	4.5±6.8 (0.3,37.6)	10.9±8.5 (3.7,37.6)

4

Supplemental Table S2 : Complete set of parameters for Mixed Meal Model as outline in STAR Methods.

Parameter	Function	Value	Source
k_1	Stomach emptying glucose (stomach $\rightarrow$ gut)	estimated	Rozendaal et al. 2018
k_2	Glucose appearance from gut (gut $\rightarrow$ plasma)	0.28	Rozendaal et al. 2018
k_3	Supression of hepatic glucose release by change in plasma glucose.	6.07x10^{-3}	Rozendaal et al. 2018
k_4	Supression of hepatic glucose release by remote insulin	2.34x10^{-4}	Rozendaal et al. 2018
k_5	Coefficient for rate of insulin dependent glucose uptake to tissues.	estimated	Rozendaal et al. 2018
k_6	Coefficient for rate of insulin production (proportion to glucose)	estimated	Rozendaal et al. 2018
k_7	Coefficient for rate of insulin production (integral term)	1.15	Rozendaal et al. 2018
k_8	Coefficient for rate of insulin production (derivative term)	7.27	Rozendaal et al. 2018
k_9	Coefficient for rate of outflow of plasma insulin to remote compartment.	3.83x10^{-2}	Rozendaal et al. 2018
k_{10}	Coefficient for rate of degradation of insulin in remote compartment.	2.84x10^{-1}	Rozendaal et al. 2018
σ	Shape factor meal	1.4	Rozendaal et al. 2018
K_m	Michaelis-Menten coefficient for glucose uptake into tissues.	13.2	Rozendaal et al. 2018
G_b	Basal glucose level (glucose set-point of model)	fasting glucose value	Maas et al. 2015
I_b	Basal insulin level (insulin set-point of model)	fasting insulin value	Maasl et al. 2015
EGP_b	Basal rate of endogenous glucose production	0.43	Rozendaal et al. 2018
f_{spill}	Fractional spill-over of LPL derived NEFA	30	O'Donovan et al. 2019
k_{11}	Coefficient of rate of LPL lipolysis of circulating triglyceride.	estimated	O'Donovan et al. 2019
ATL_{max}	Coefficient for maximum rate of lipolysis of triglyceride stored in adipose tissue.	0.215	O'Donovan et al. 2019
K_{ATL}	Michaelis-Menten coefficient for rate of lipolysis of adipose triglyceride	estimated	O'Donovan et al. 2019
k_{12}	Coefficient for rate of NEFA uptake into tisues	estimated	new
τ_{LPL}	Time delay coefficient for insulin effect on lipid reactions.	estimated	O'Donovan et al. 2019
k_{13}	Stomach emptying triglyceride (stomach $\rightarrow$ gut)	8.8x10^{-3}	new
k_{14}	Rate constant for triglyceride appearance in plasma (gut $\rightarrow$ lymphatic compartment $\rightarrow$ plasma)	estimated	new
k_{15}	Coefficient for inhibition of triglyceride secretion from liver by delayed insulin.	1.0x10^{-5}	new
k_{16}	Basal rate of triglyceride secretion from liver (VLDL)	estimated	new

Supplemenary Section S1: Complete set of equations for Mixed Meal Model Introduced in STAR Methods

Equation	Role	Source
**Glucose**		
$G_{meal} = \sigma k_1^{\sigma} t^{\sigma-1} e^{-k_1 t^{\sigma}} \cdot D_G$	Glucose mass in stomach	Maas et al. 2015
$\frac{d[\boldsymbol{M_{G-gut}}]}{dt} = G_{meal} - k_2[\boldsymbol{M_{G-gut}}]$	Rate of transition of glucose from stomach through gut to plasma.	Rozendaal et al. 2018
$G_{gut} = k_2(\frac{f_G}{V_G \cdot BW})[\boldsymbol{M_{G-gut}}]$	Glucose appearance in plasma from the meal via the gut.	Rozendaal et al. 2018
$G_{liver} = EGP_b - k_4[\boldsymbol{I_{d1}}] - k_3([\boldsymbol{G_{PL}}] - G_b)$	Net hepatic glucose flux - EGP inhibited by insulin and glucose	Rozendaal et al. 2018
$G_{uii} = EGP_b(\frac{K_m+G_b}{G_b}) \cdot (\frac{[\boldsymbol{G_{PL}}]}{K_m+[\boldsymbol{G_{PL}}]})$	Insulin independent glucose uptake into tissues (maintain steady state)	Rozendaal et al. 2018
$G_{uid} = k_5[\boldsymbol{I_{d1}}](\frac{[\boldsymbol{G_{PL}}]}{K_M+[\boldsymbol{G_{PL}}]})$	Insulin dependent glucose uptake into tissues (delayed insulin signal)	Rozendaal et al. 2018
$G_{ren} = (\frac{c_1}{V_G \cdot BW})([\boldsymbol{G_{PL}}] - G_{ren})([\boldsymbol{G_{PL}}] > G_{ren})$	Renal excretion of excess glucose (iff $G_{PL} >$ sepecified threshold)	Rozendaal et al. 2018
$\frac{d[\boldsymbol{G_{PL}}]}{dt} = G_{gut} + G_{liver} - G_{uii} - G_{uid} - G_{ren}$	Rate of change of plasma glucose	Rozendaal et al. 2018
**Insulin**		
$I_{pro} = k_6([\boldsymbol{G_{PL}}] - G_b) + \frac{k_7}{\tau_i}(G_{int} + G_b) + \frac{k_8}{\tau_d}(\frac{d[\boldsymbol{G_{PL}}]}{dt})$	Insulin production in pancreas (PID controller)	Rozendaal et al. 2018
$I_{liver} = k_7(\frac{G_b}{\tau_i \cdot I+b})[\boldsymbol{I_{PL}}]$	Insulin degradation in liver (maintain steady state)	Rozendaal et al. 2018
$I_{rem} = k_9([\boldsymbol{I_{PL}}] - I_b)$	Insulin transport to insterstitial space	Rozendaal et al. 2018
$\frac{d[\boldsymbol{I_{PL}}]}{dt} = I_{pro} - I_{liver} - I_{rem}$	Rate pf change of plasma insulin	Rozendaal et al. 2018
$\frac{d[\boldsymbol{I_{d1}}]}{dt} = k_9([\boldsymbol{I_{Pl}}]I_b) - k_{10} \cdot [\boldsymbol{I_{d1}}]$	Insulin delay 1 (glucose)	Rozendaal et al. 2018
$\frac{d[\boldsymbol{I_{d2}}]}{dt} = \frac{3}{\tau_{LPL}}([\boldsymbol{I_{PL}}] - [\boldsymbol{I_{d2}}])$	Insulin delay 2 (triglyceride liver)	O'Donovan et al. 2019
$\frac{d[\boldsymbol{I_{d3}}]}{dt} = \frac{3}{\tau_{LPL}}([\boldsymbol{I_{d2}}] - [\boldsymbol{I_{d3}}])$	Insulin delay 3	O'Donovan et al. 2019
$\frac{d[\boldsymbol{I_{d4}}]}{dt} = \frac{3}{\tau_{LPL}}([\boldsymbol{I_{d3}}] - [\boldsymbol{I_{d4}}])$	Insulin delay 4 (LPL lipolysis)	O'Donovan et al. 2019

6

Equation	Role	Source
**Triglyceride**		
$TG_{meal} == \sigma k_{13}^{\sigma} t^{\sigma-1} e^{-k_{13}t^{\sigma}} \cdot D_{TG}$	Triglyceride mass in stomach	[new]
$\frac{d[\boldsymbol{M_{TG-gut1}}]}{dt} = TG_{meal} - k_{14} \cdot [\boldsymbol{M_{TG-gut1}}]$	delayed transition of triglyceride mass from stomach to plasma via lymphatic	[new]
$\frac{d[\boldsymbol{M_{TG-gut2}}]}{dt} = k_{14}([\boldsymbol{M_{TG-gut2}}] - [\boldsymbol{M_{TG-gut1}}])$	system. (gut → lymphatic system → plasma)	[new]
$\frac{d[\boldsymbol{M_{TG-gut3}}]}{dt} = k_{14}([\boldsymbol{M_{TG-gut3}}] - [\boldsymbol{M_{TG-gut2}}])$		[new]
$TG_{gut} = k_{15}(\frac{f_{TG}}{V_{TG} \cdot BW} \cdot [\boldsymbol{M_{TG-gut3}}]$	Triglyceride appearance in plasma from the meal via the lymphatic system.	[new]
$TG_{LPL} = k_{11} \cdot [\boldsymbol{TG_{PL}}] \cdot [\boldsymbol{I_{d4}}]$	Hydrolysis of circulating triglcyeride by LPL (Insulin stimulated)	O'Donovan et al. 2019
$TG_{VLDL} = k_{16} - k_{15}([\boldsymbol{I_{d4}}] - I_b)$	Secretion of triglyceride from the liver occurs at a basal rate and inhibited by insulin.	[new]
$\frac{d[\boldsymbol{TG_{PL}}]}{dt} = TG_{VLDL} + TG_{gut} - TG_{LPL}$	Rate of change of plasma triglyceride concentration.	[new]
**NEFA**		
$\frac{d[\boldsymbol{NEFA_{PL}}]}{dt} = 3spill \cdot [\boldsymbol{TG_{PL}}] + \frac{ATL_{max}}{1+K_{ATL} \cdot [\boldsymbol{I_{d2}}]^2} - k_{12}[\boldsymbol{NEFA_{PL}}]$	Rate of change of plasma NEFA concentration.	[new]

7

Supplemenary Section S2: Results of local parameter sensitivity analysis described in STAR Methods

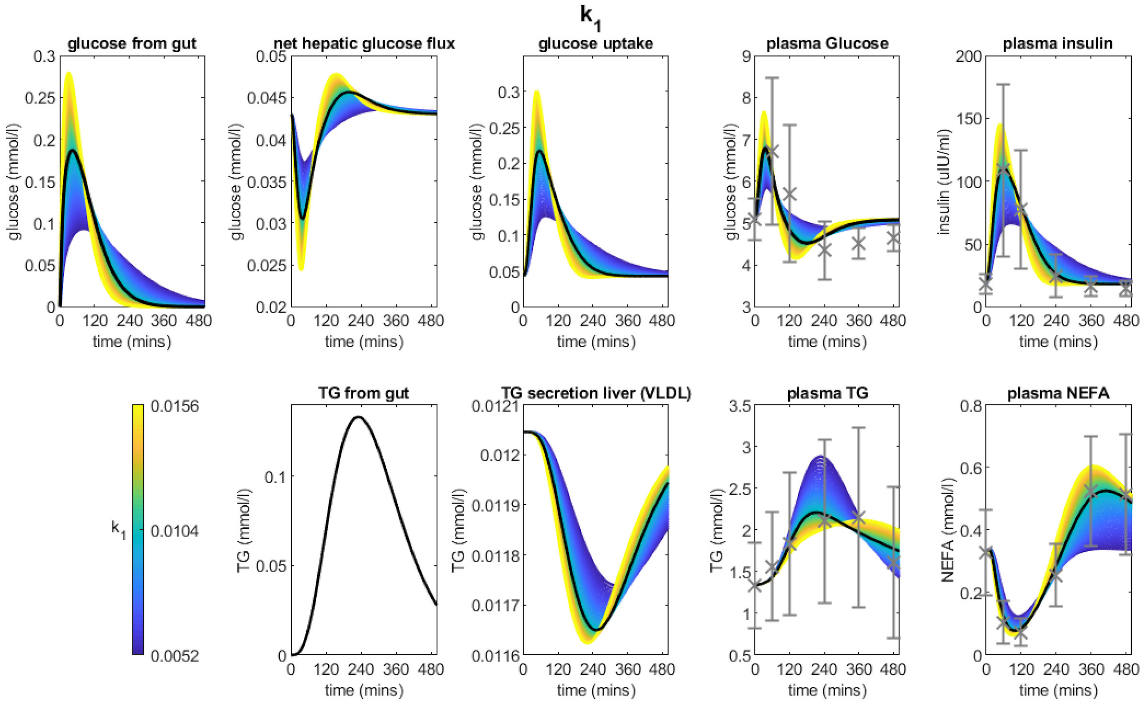

Supplementary Figure S4 : Local parameter sensitivity analysis for k_1 - coefficient for rate of stomach emptying for glucose in meal

50% variation in the estimated value for k_1 has a substantial impact on all Meal Model fluxes. Thereforek_1 is deemed a sensitivie model parameter and is estimate from data.

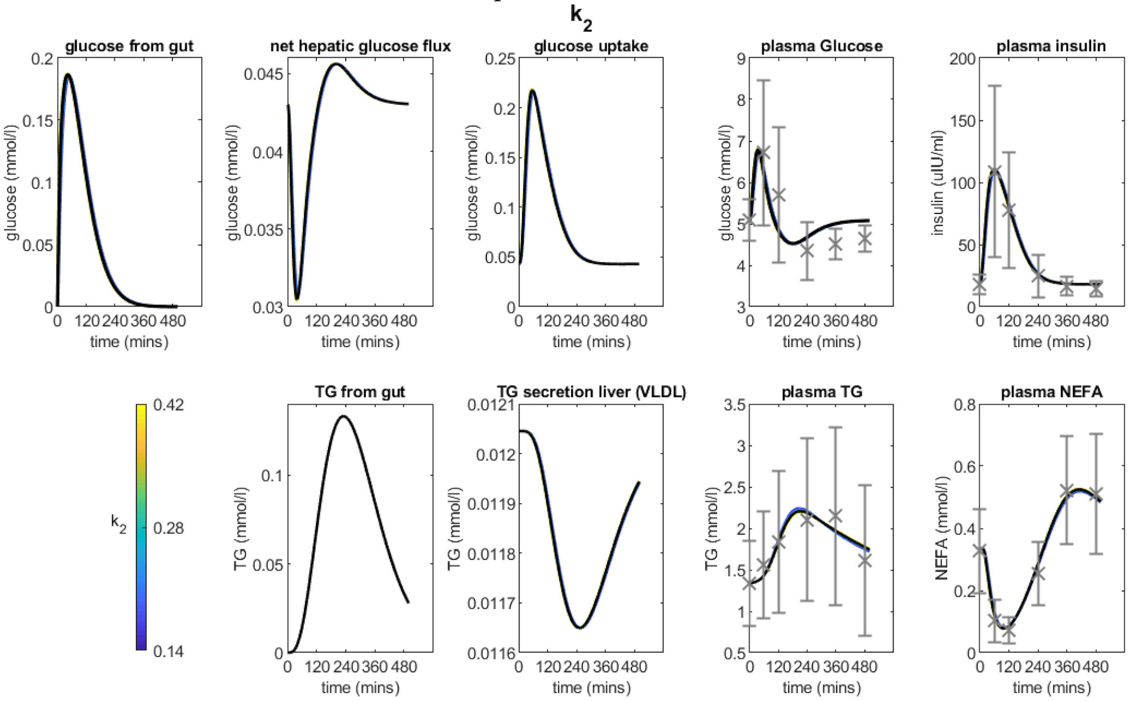

Supplementary Figure S5 : Local parameter sensitivity analysis for k_2 - coefficient for rate of appearance of glucose from the gut

50% variation in the estimated value for k_2 has little effect on the Meal Model fluxes. Thereforek_2 is kept fixed to 0.28, the value proposed by Rozendaal et al (2018) for our analyses.

8

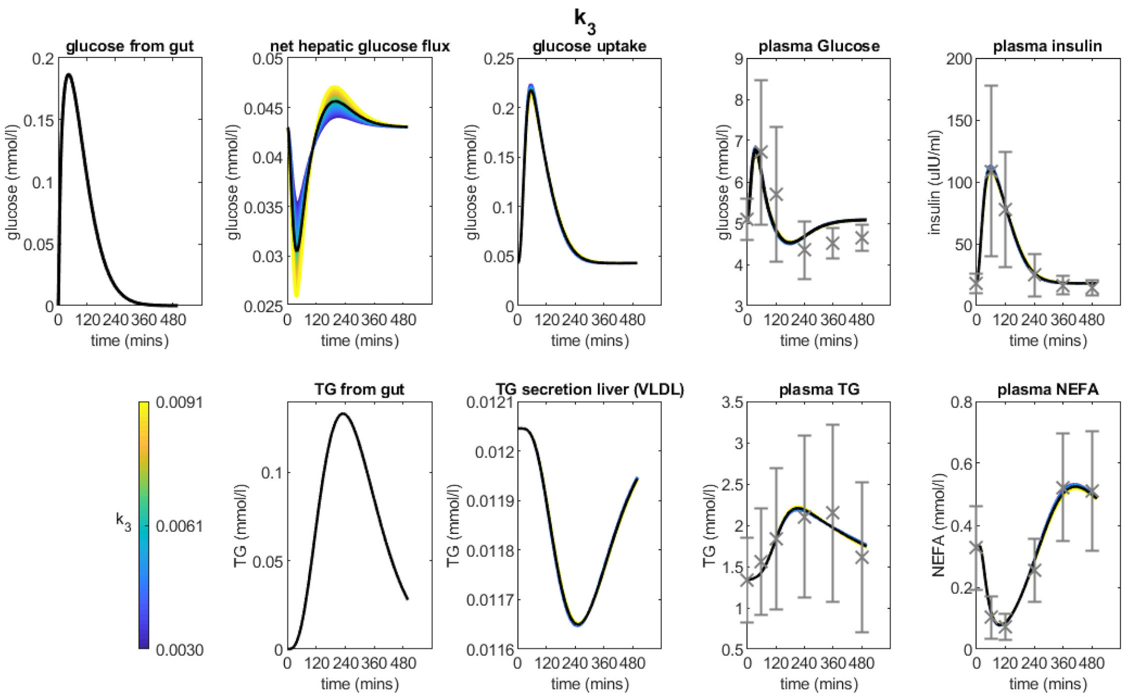

Supplementary Figure S6: Local parameter sensitivity analysis for k_3 - coefficient for the rate of suppression of endogenous glucose production by plasma glucose
While a 50% variation in the estimated value for k_3 alters the model predicted net hepatic glucose flux, it has little effect on the remaining Meal Model fluxes. Therefore, for our analyses k_3 is kept fixed to 6.07×10^{-3} in line with the value proposed by Rozendaal et al (2018).

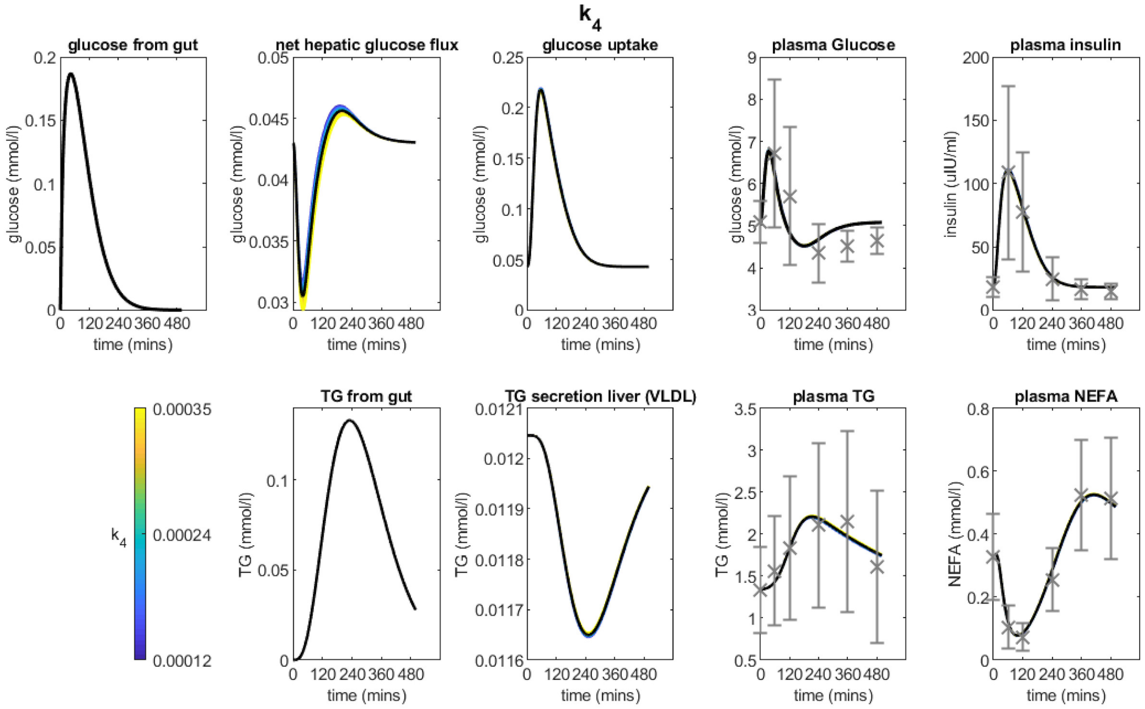

Supplementary Figure S7: Local parameter sensitivity analysis for k_4 - coefficient for the rate of suppression of endogenous glucose production by remote insulin
While a 50% variation in the estimated value for k_4 has a very modest effect on the model predicted net hepatic glucose flux. Therefore, for our analyses k_4 is kept fixed to 2.35×10^{-4} in line with the value proposed by Rozendaal et al (2018).

9

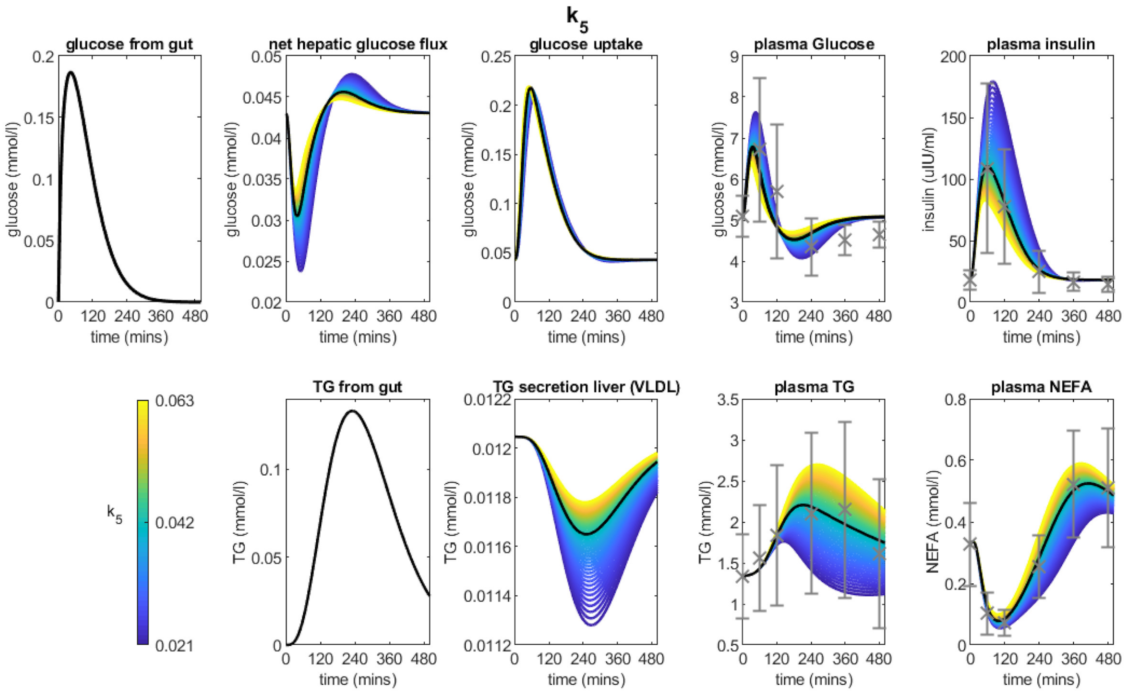

Supplementary Figure S8: Local parameter sensitivity analysis for k_5 - coefficient for rate of insulin mediate glucose uptake into tissues

A 50% variation in the estimated value for k_5 has a modest effect on rate of glucose uptake into the tissues, altering the plasma glucose concentrations. which in turn has a knock-on effect on other Meal Model fluxes is . As a result, k_5 is deemed a sensitivie model parameter and is estimated from data.

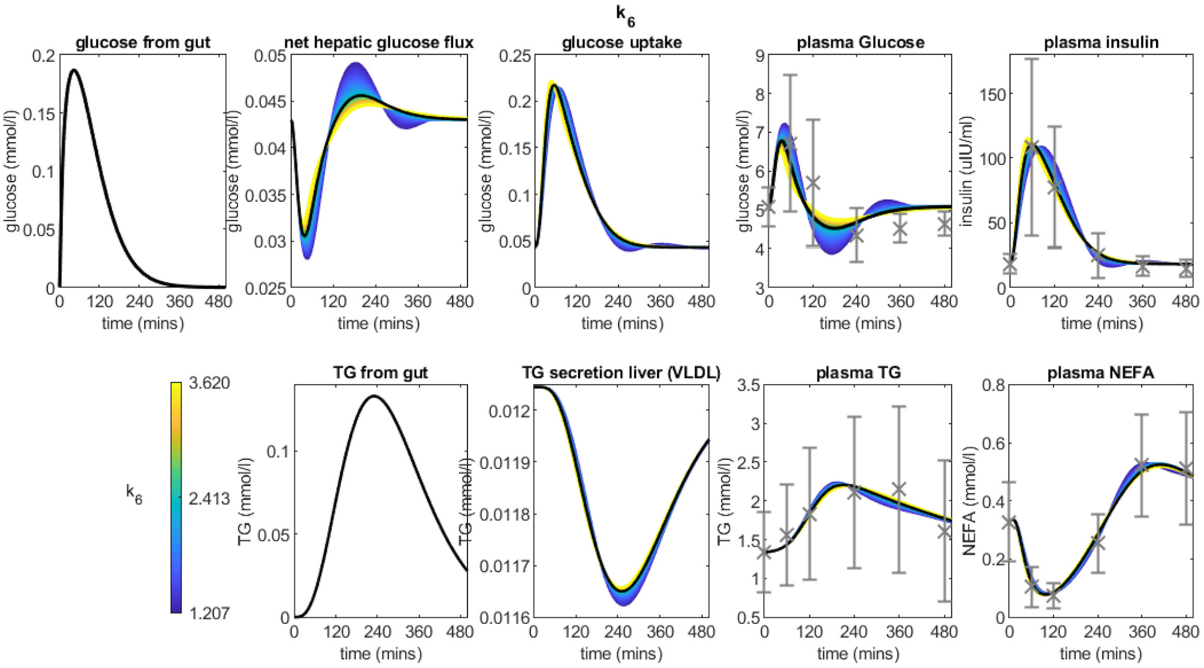

Supplementary Figure S9: Local parameter sensitivity analysis for k_6 - coefficient rate of insulin secretion (proportional term).

A 50% variation in the estimated value for k_6 alters the rate of insulin secretion . As a result, k_6 is deemed a sensitivie model parameter and is estimated from data.

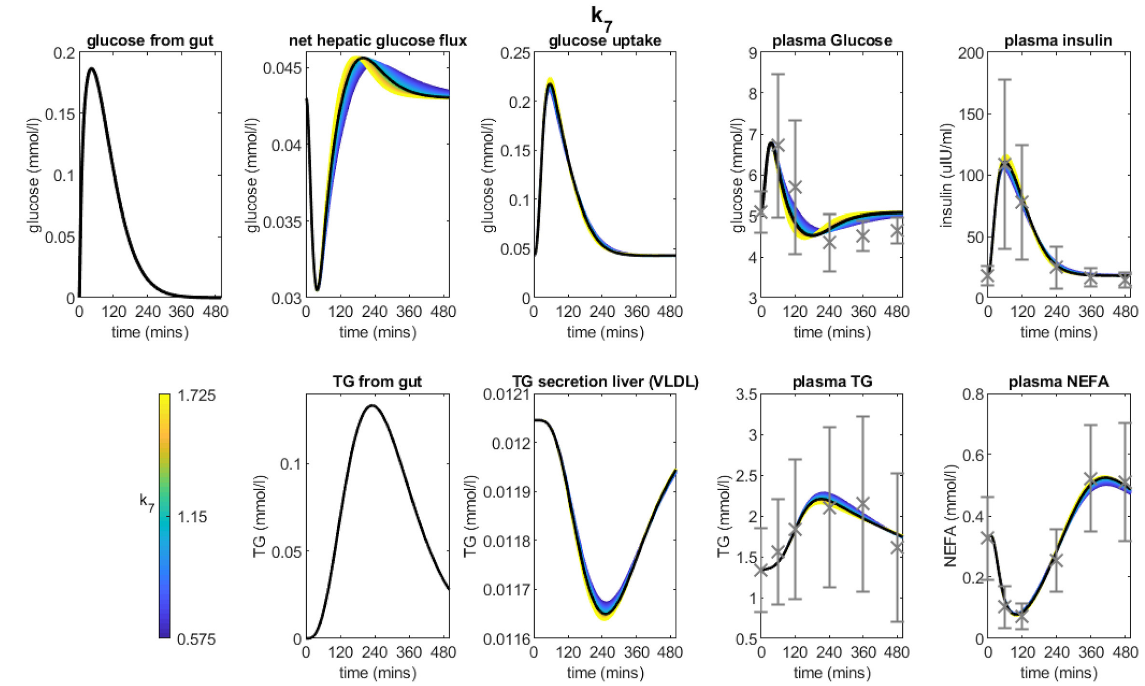

Supplementary Figure S10: Local parameter sensitivity analysis for k_7 - coefficient rate of insulin secretion (integral term).

A 50% variation in the estimated value for k_7 results in modest alterations in the other Meal Model fluxes is. Consequently, k_7 is kept fixed to 1.15 in line with the value proposed by Rozendaal et al (2018).

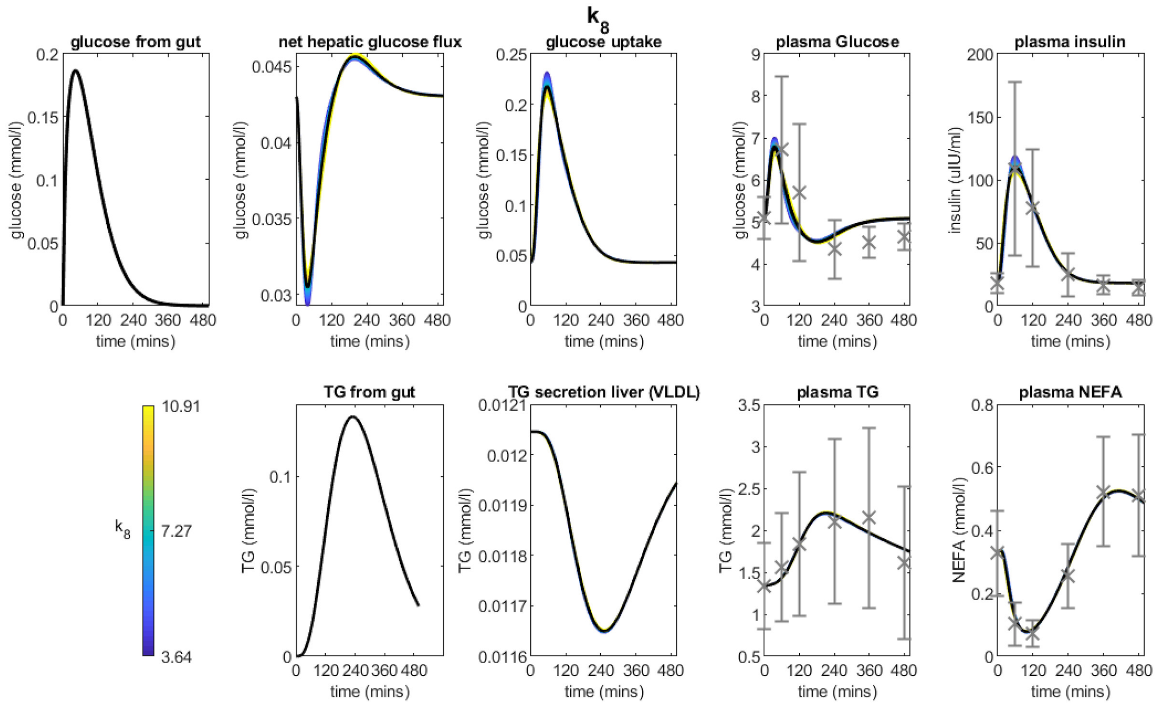

Supplementary Figure S11: Local parameter sensitivity analysis for k_8 - coefficient rate of insulin secretion (derivativel term).

A 50% variation in the estimated value for k_8 has little effect on M^{3}al Model fluxes. Consequently, k_8 is kept fixed to 7.27 in line with the value proposed by Rozendaal et al (2018).

11

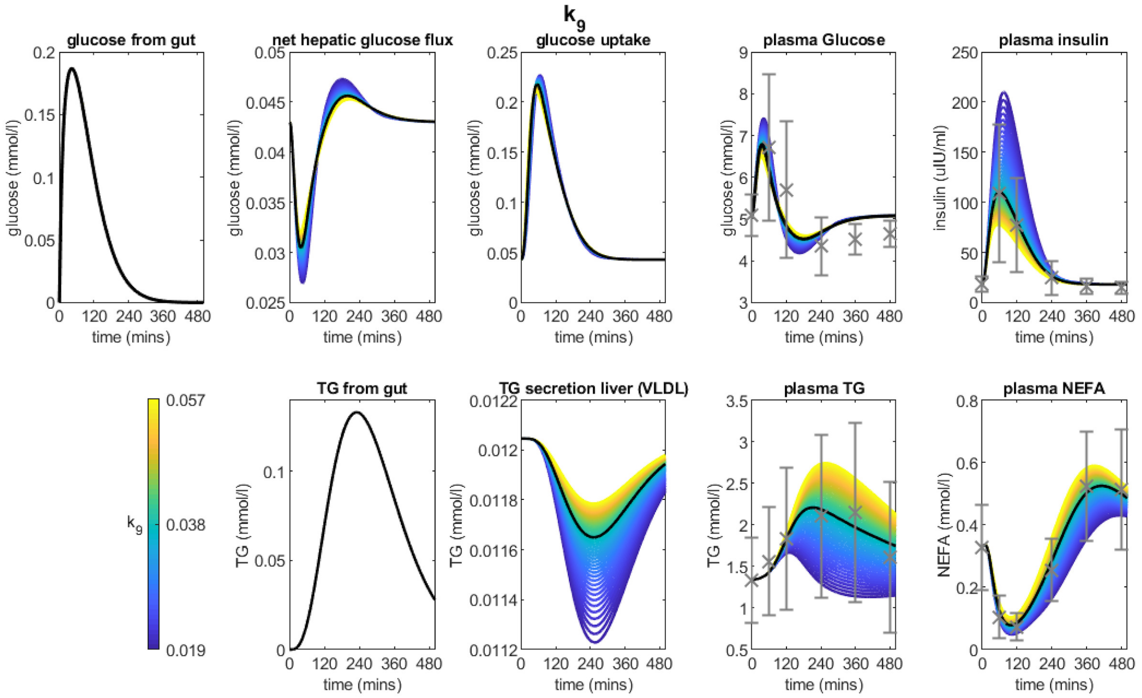

Supplementary Figure S12: Local parameter sensitivity analysis for k_9 - coefficient rate of outflow of insulin to intersititial space.
A 50% variation in the estimated value for k_9 alters the effect of insulin of glucose uptake into the periphery, affecting the plasma glucose concentration and thereby all of the other Meal Model fluxes by altering the rate of insulin section. While k_9 is a senstive parameter, it can be difficult to estimate reliably from plasma data. Consequently, k_9 is kept fixed to 3.83×10^{-2} in line with the value proposed by Rozendaal et al (2018).

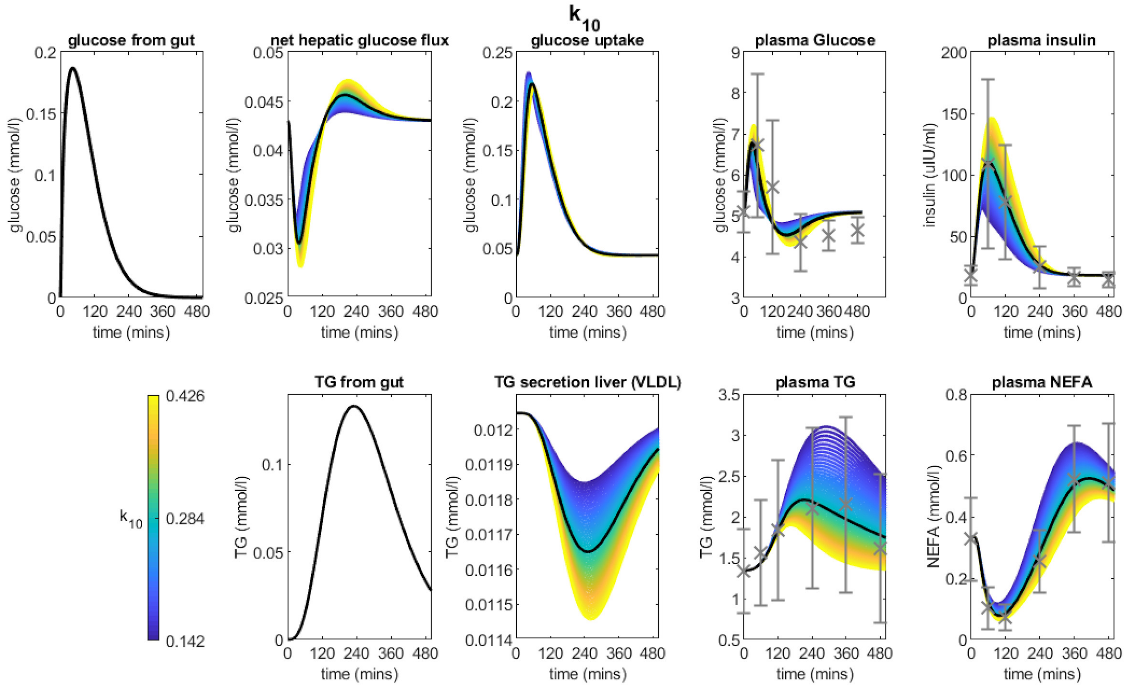

Supplementary Figure S13: Local parameter sensitivity analysis for k_{10} - coefficient rate of degradation of insulin in intersititial compartment.
A 50% variation in the estimated value for k_{10} alters the other Meal Model fluxes. As done with k_9, k_{10} is kept fixed to 0.284 in line with the value proposed by Rozendaal et al (2018).

12

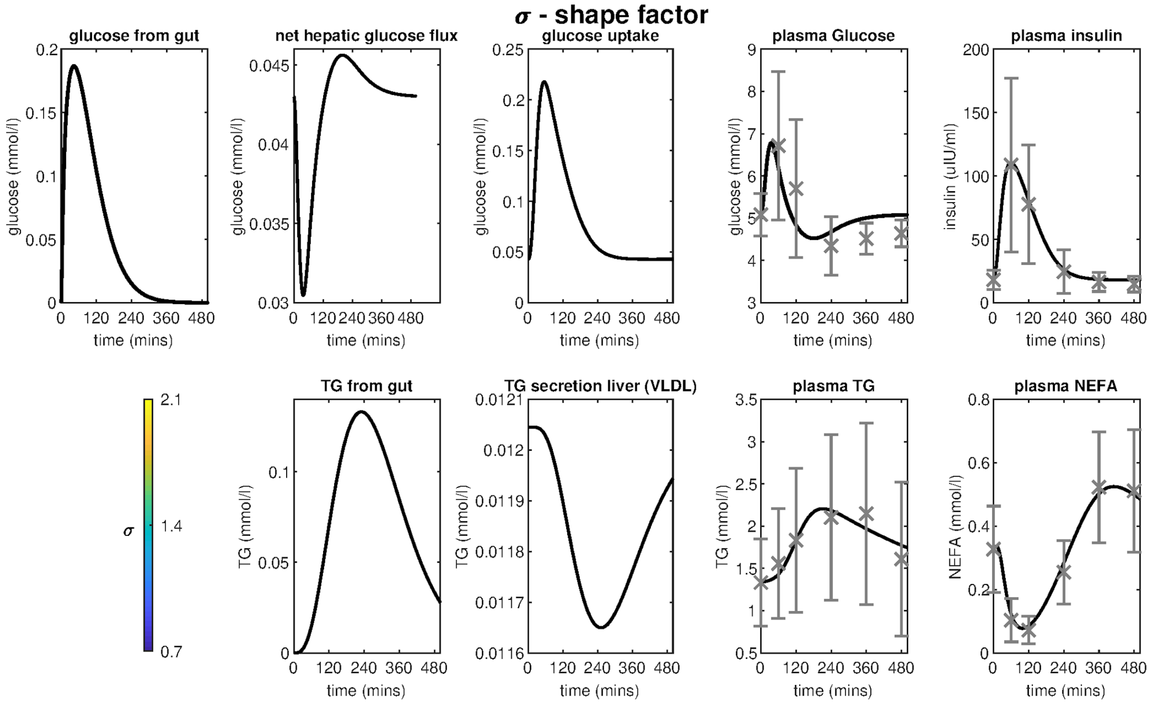

Supplementary Figure S14: Local parameter sensitivity analysis for σ - shape factor for glucose rate of appearance from meal.
A 50% variation in the estimated value for σ has not obvious effect on the Meal Model fluxes. Consequently, σ is kept fixed to 1.4 in line with the value proposed by Rozendaal et al (2018).

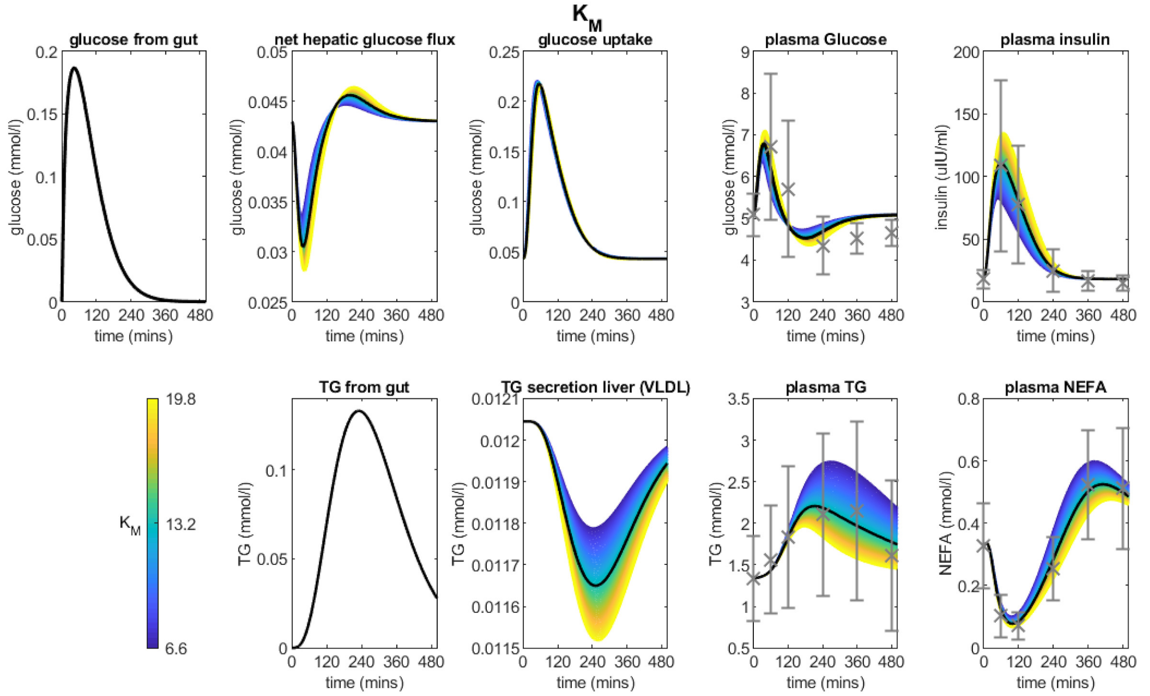

Supplementary Figure S15: Local parameter sensitivity analysis for K_M - Michaelis Menten coefficient for glucose uptake into tissues.
A 50% variation in the estimated value for K_M alters the rate of glucose uptake into the tissues, which in turn alters the plasma glucose concentation and from there the other Meal Model fluxes are changed. However, biologically we are not interested in changes in K_M. Consequently, K_M is kept fixed to 13.2 in line with the value proposed by Rozendaal et al (2018).

13

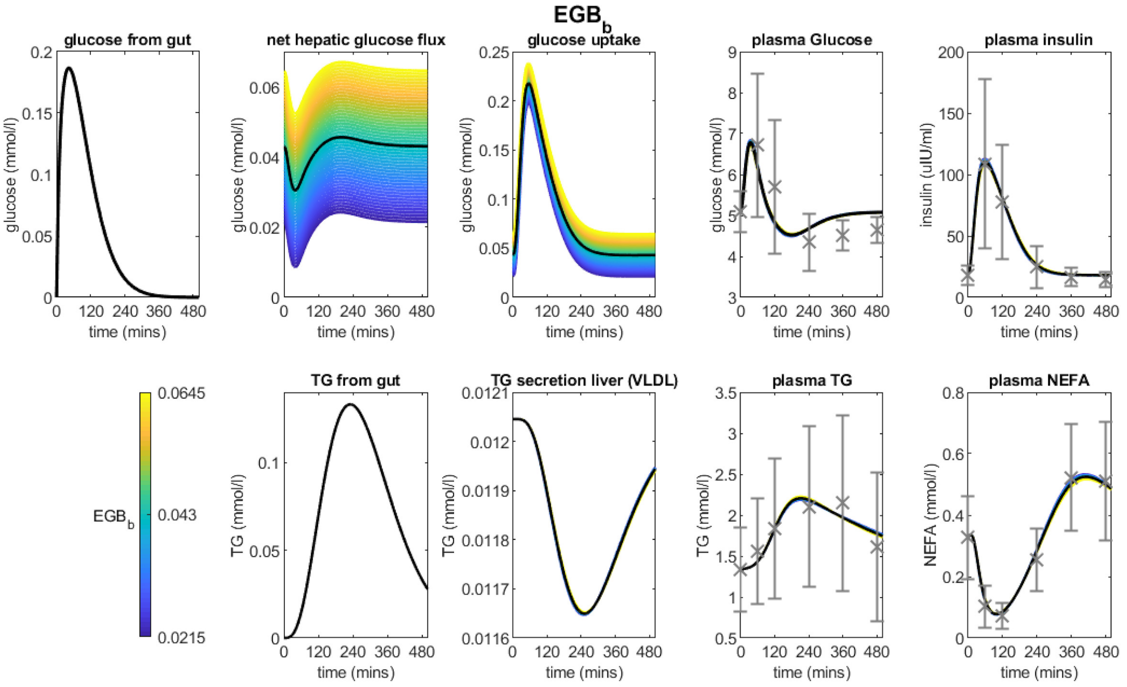

Supplementary Figure S16: Local parameter sensitivity analysis for EGP_b - basal rate of endogenous glucose production.
A 50% variation in the estimated value for EGP_b shifts the fasting level of the net hepatic glucose flux, which is compensated by the rate of glucose uptake into tissues thereby having no impact on the other Meal Model fluxes. For this reason, EGP_b is kept fixed to 0.043 in line with the value proposed by Rozendaal et al (2018).

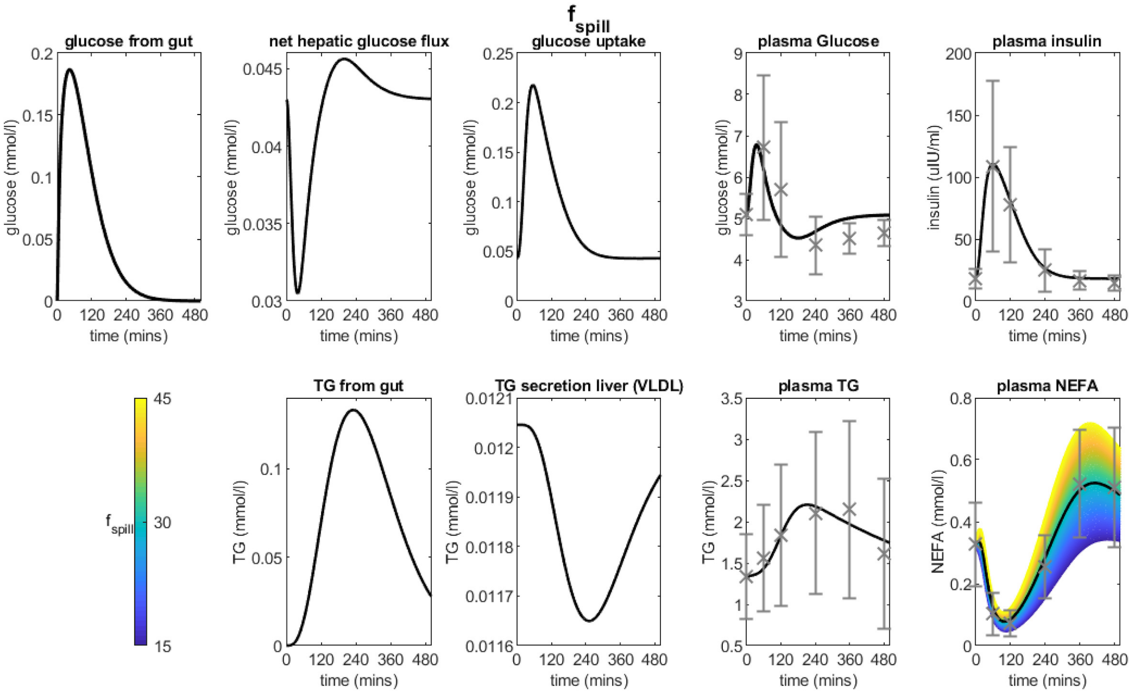

Supplementary Figure S17: Local parameter sensitivity analysis for f_{spill} - fractional spillover of LPL derived NEFA into plasma.
A 50% variation in the estimated value for f_{spill} alters the plasma NEFA concentration, however has no effect on the other Meal Model fluxes. Consequently, f_{spill} is fixed to 30.

14

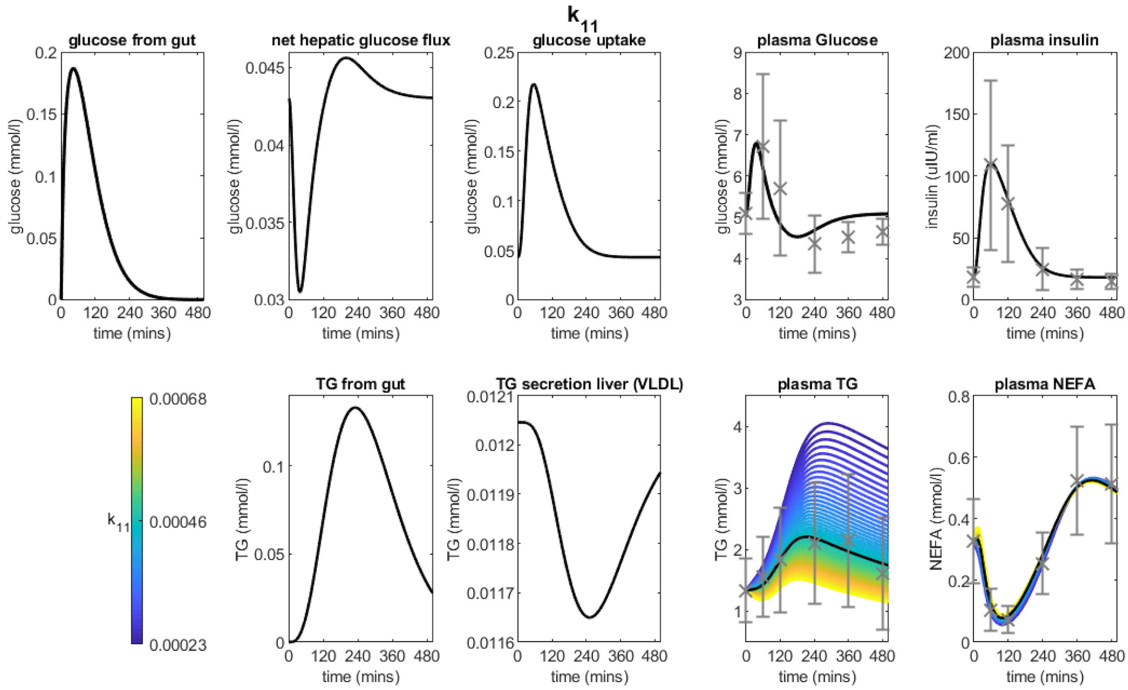

Supplementary Figure S18: Local parameter sensitivity analysis for k_{11} - coefficient for rate of LPL lipolysis of circulating triglyceride.
A 50% variation in the estimated value for k_{11} has a substantial impact on the plasma triglyceride concentration, which in turn results in modest alterations in the plasma NEFA concentration. As a result, k_{11} is deemed a sensitivie model parameter and is estimated from data.

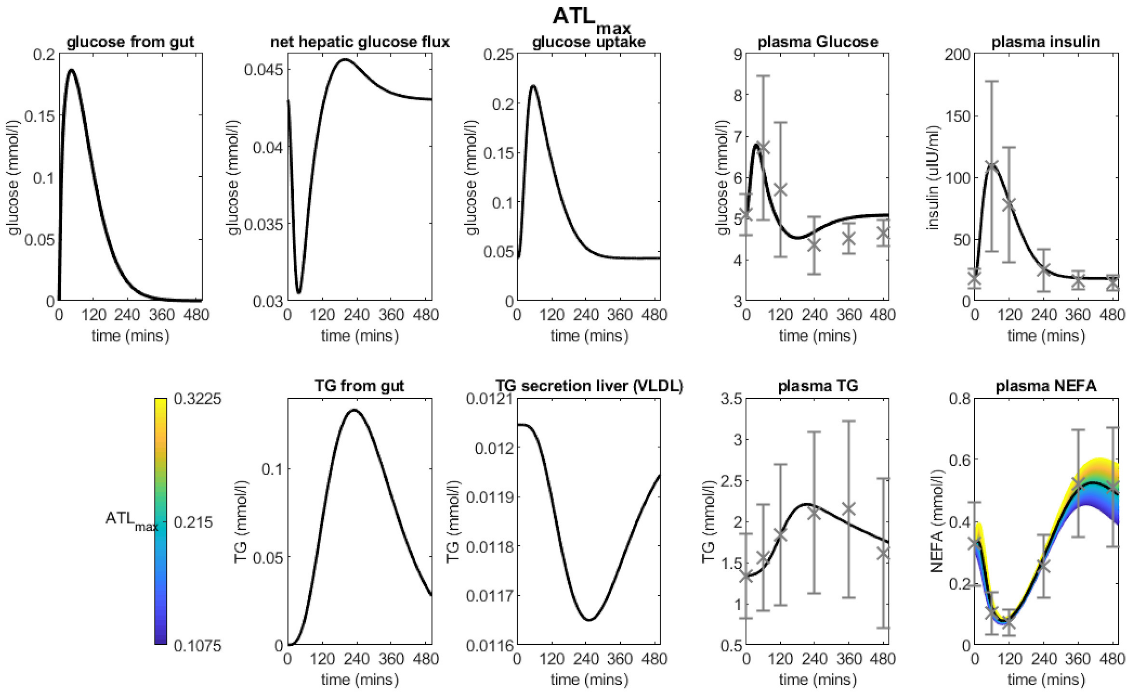

Supplementary Figure S19: Local parameter sensitivity analysis for ATL_{max} - coefficient for maximum rate of lipolysis of triglyceride stored within the adipose tissue.
A 50% variation in the estimated value for ATL_{max} has an effect on the plasma NEFA concentration. However, due to issuess with identifiability ATL_{max} is fixed to 0.215 (estimated by fitting to average meal response NutriTecht).

15

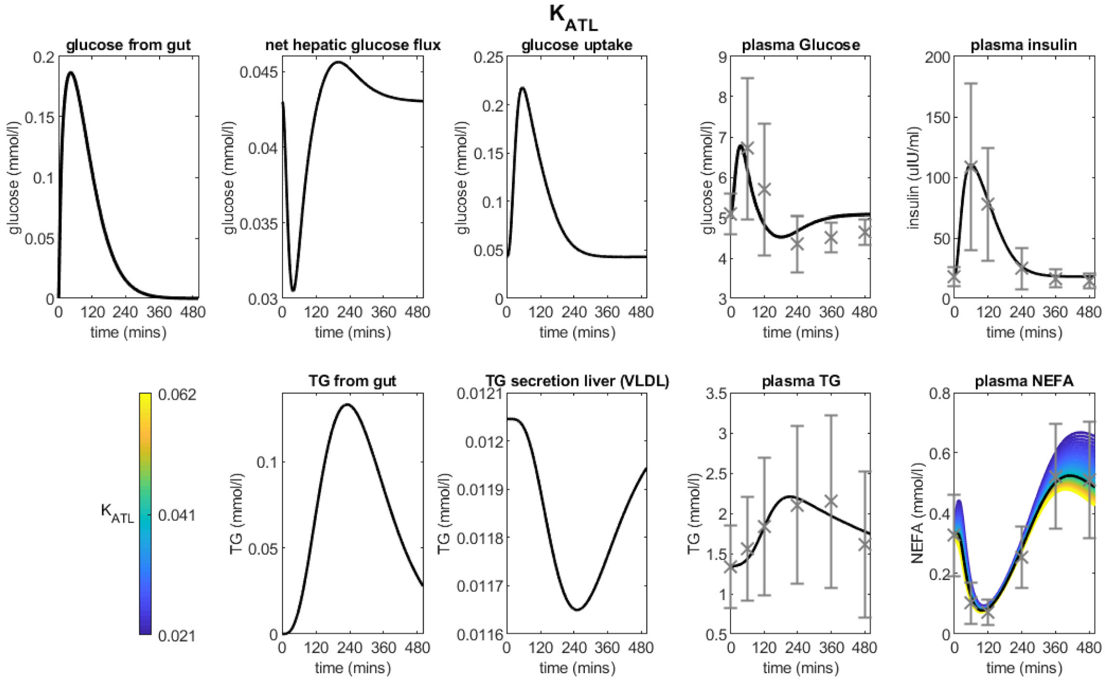

Supplementary Figure S20: Local parameter sensitivity analysis for K_{ATL} - Michaelis Menten coefficient for rate of lipolysis of triglyceride stored within the adipose tissue.
A 50% variation in the estimated value for K_{ATL} has a substantial impact on the plasma NEFA concentration. As a result, K_{ATL} is deemed a sensitivie model parameter and is estimated from data.

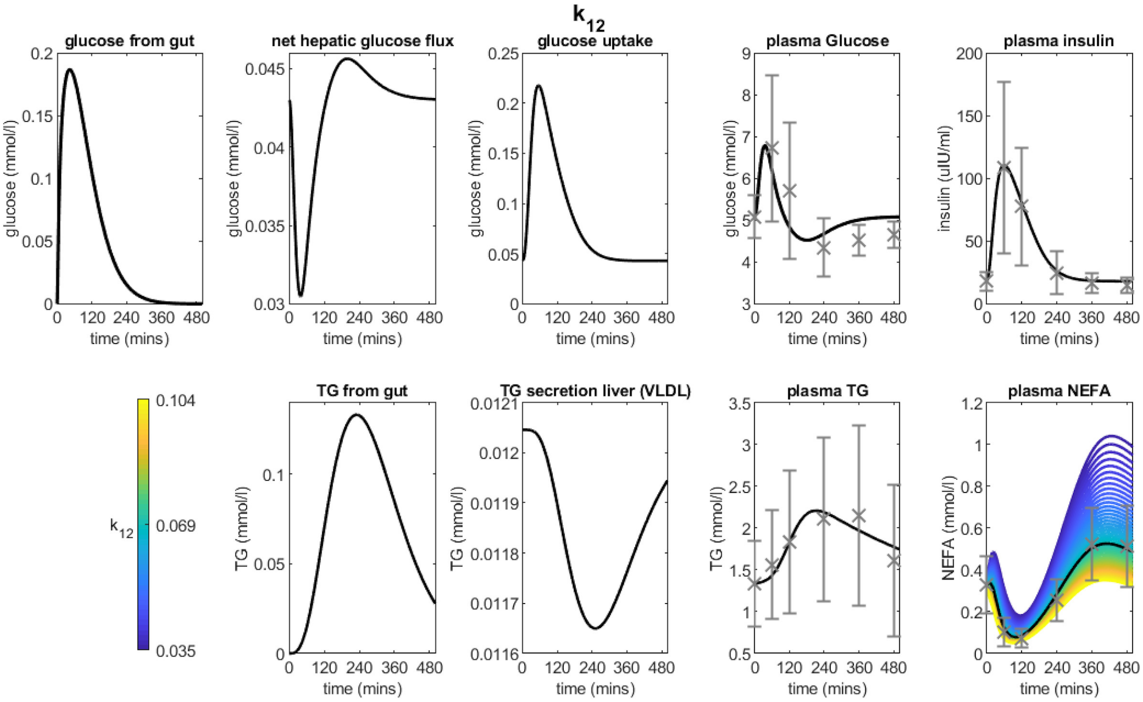

Supplementary Figure S21: Local parameter sensitivity analysis for k_{12} - coefficient for rate of NEFA uptake into tissues.
A 50% variation in the estimated value for k_{12} has a substantial impact on the plasma NEFA concentration. As a result, k_{12} is deemed a sensitivie model parameter and is estimated from data.

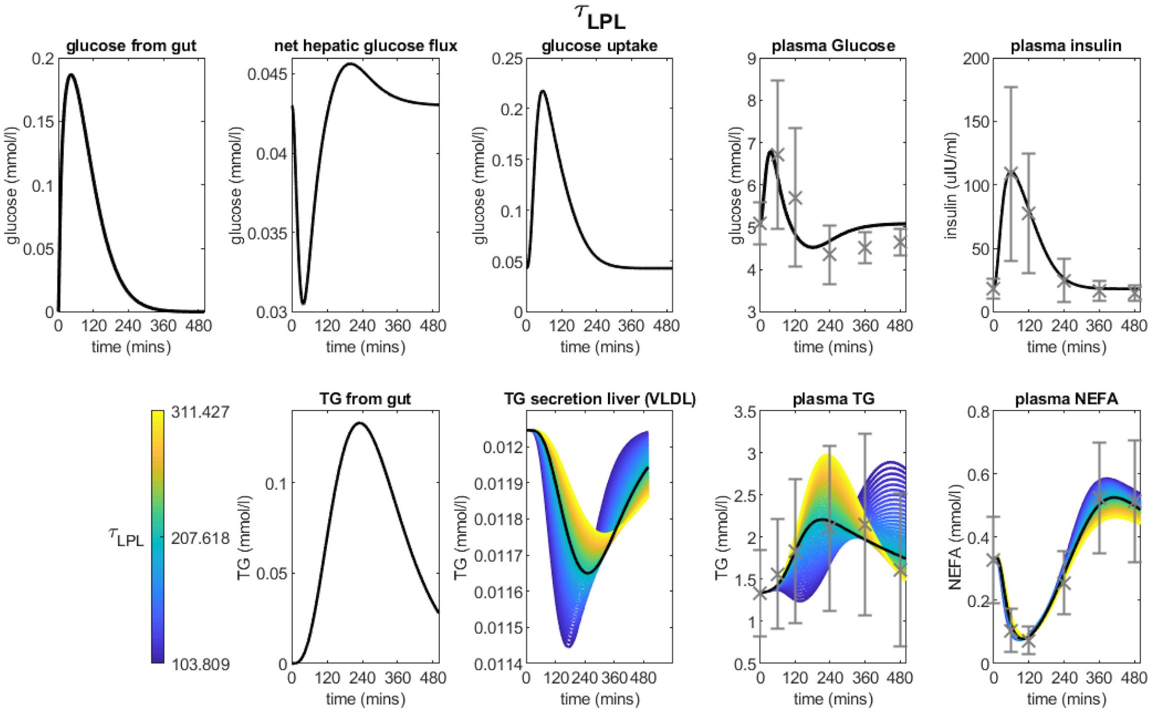

Supplementary Figure S22: Local parameter sensitivity analysis for τ_{LPL} - time delay coefficient for effect of insulin on lipid reactions.
A 50% variation in the estimated value for τ_{LPL} has a substantial impact on all lipid fluxes within the Meal Model. As a result, τ_{LPL} is deemed a sensitivie model parameter and is estimated from data.

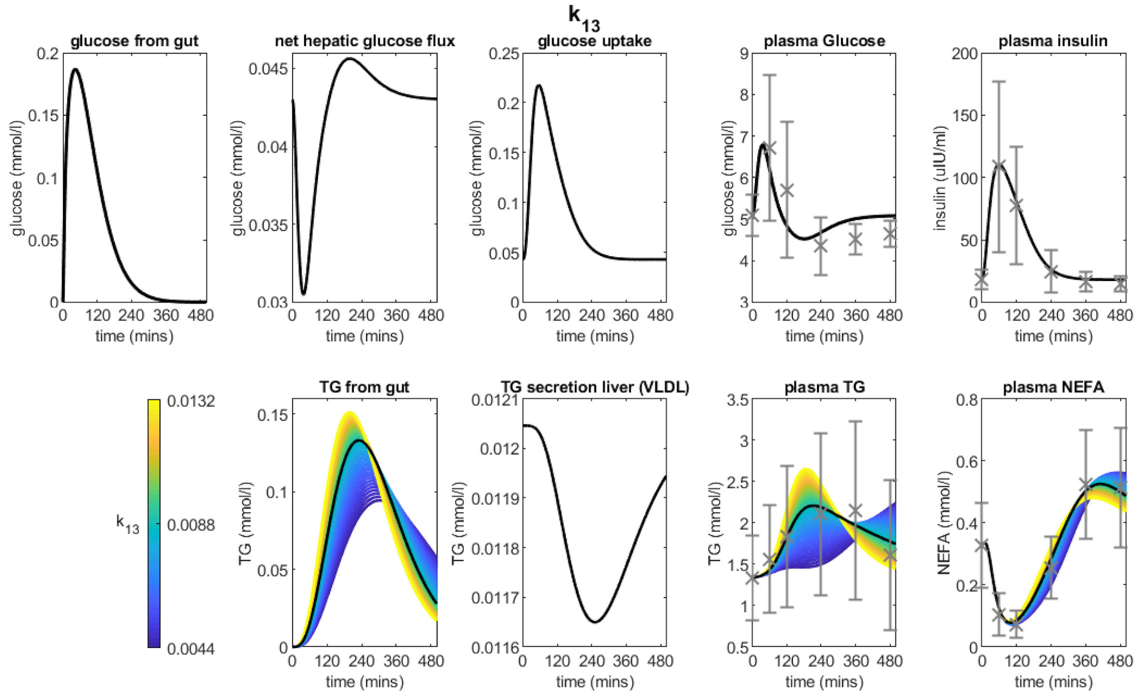

Supplementary Figure S23: Local parameter sensitivity analysis for k_{13} - coefficient for rate of stomach empyting for triglyceride in meal.
A 50% variation in the estimated value for k_{13} has a substantial impact on the rate of appearance of triglyceride, which in turn impacts both the plasma NEFA and triglyceride concentrations. However, due to to identifiability issues when estimated with k_{14} k_{13} is fixed to 0.0088 (values estimated for the average meal response from the NutriTech Study).

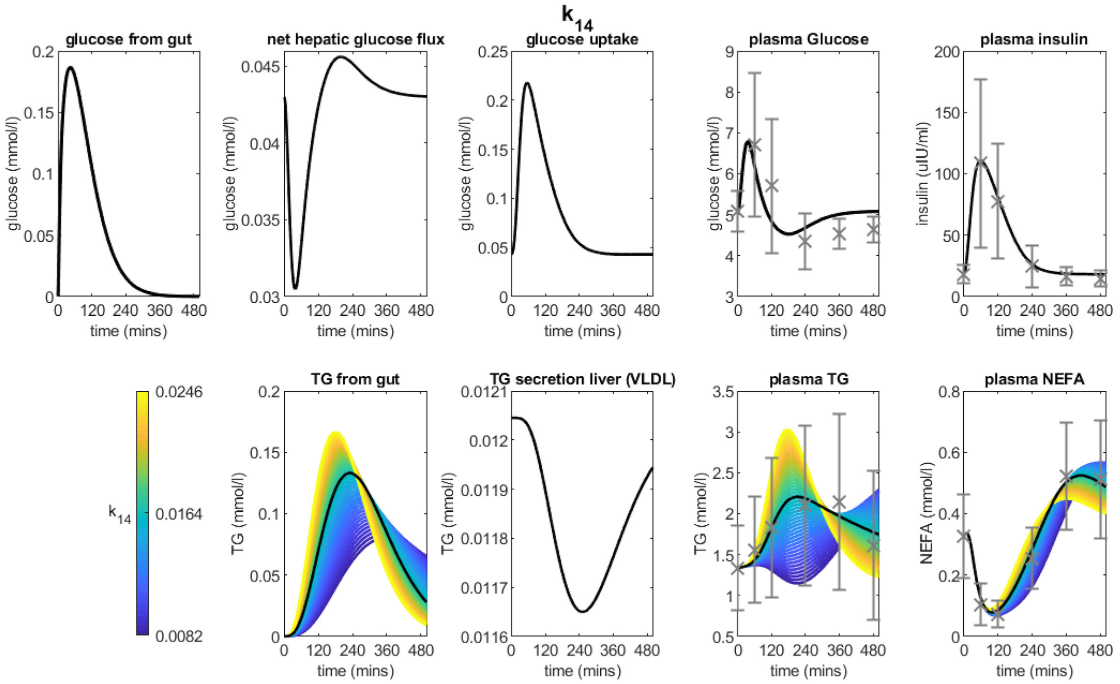

Supplementary Figure S24: Local parameter sensitivity analysis for k_{14} - coefficient for rate of triglyceride appearance from gut via lymphatic system.
A 50% variation in the estimated value for k_{14} has a substantial impact on the rate of appearance of triglyceride, which in turn impacts both the plasma NEFA and triglyceride concentrations. As a result, k_{14} is deemed a sensitivie model parameter and is estimated from data.

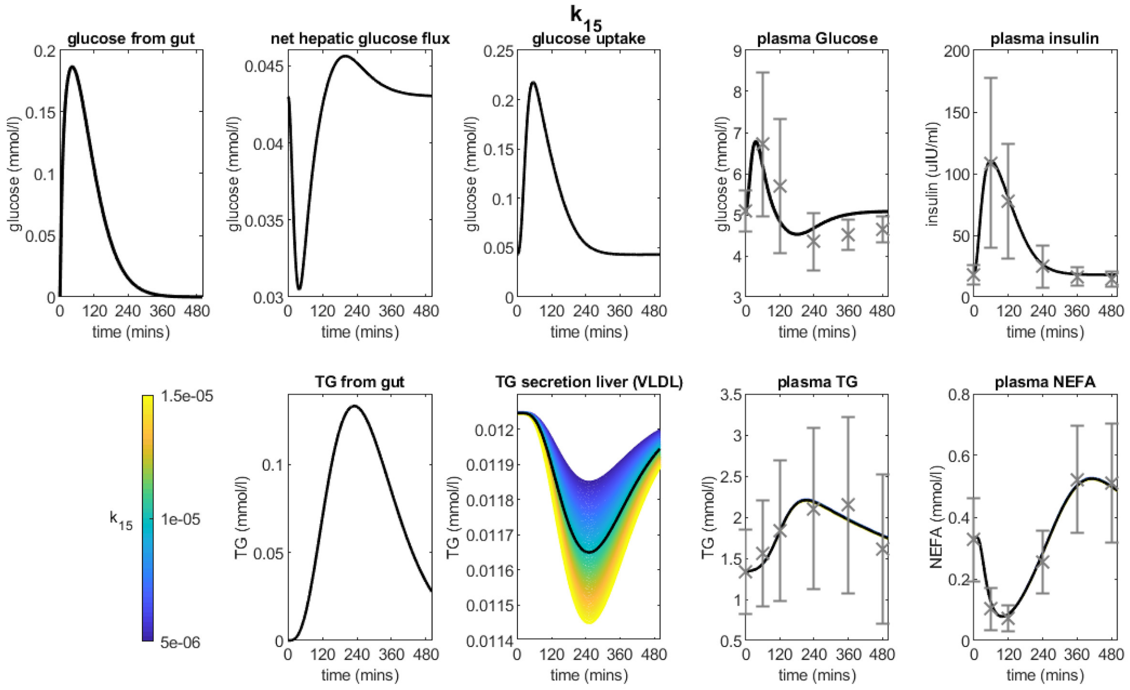

Supplementary Figure S25: Local parameter sensitivity analysis for k_{15} - coefficient for the inhibition of endogenous triglyceride secretion by insulin.
A 50% variation in the estimated value for k_{15} has a substantial impact on the rate of triglyceride secretion from the liver, however this is compenstated for by the rate of LPL lipolysis and has no impact on the resulting plasma tirglyceride concentration. Moreover, k_{15} is not identifiable. Consequently, k_{15} is fixed to 1×10^{-5}.

18

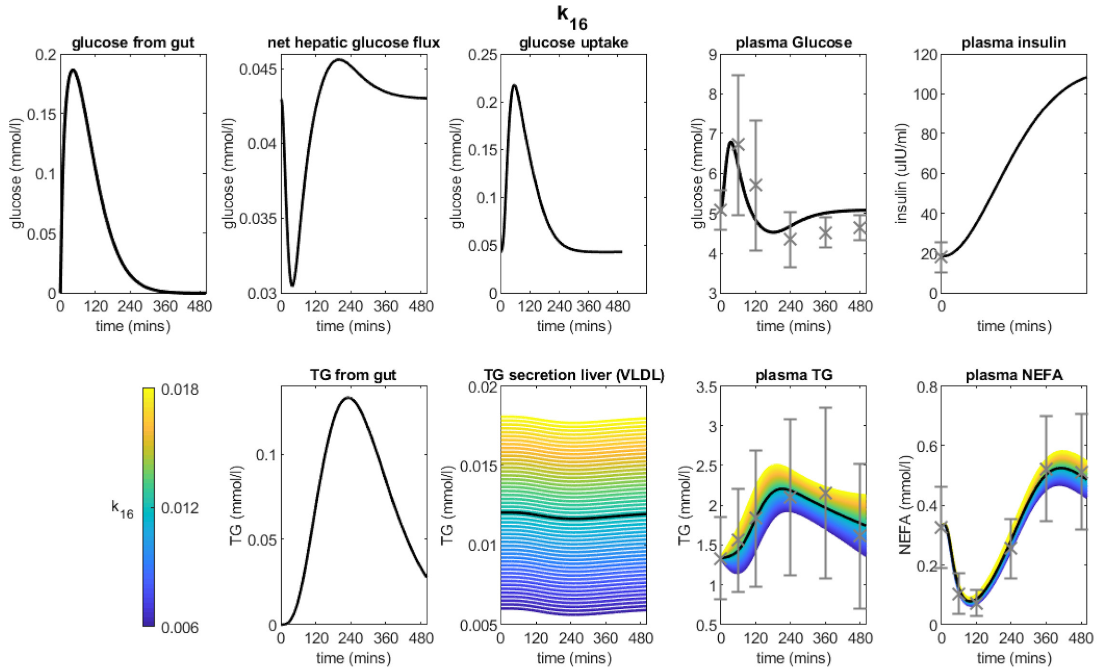

Supplementary Figure S26: Local parameter sensitivity analysis for k_{16} - basal rate of secretion of endogenous triglyceride from the liver.
A 50% variation in the estimated value for k_{16} has a substantial impact on the rate of triglyceride secretion from the liver, which in turn impacts both the plasma NEFA and triglyceride concentrations. As a result, k_{16} is deemed a sensitivie model parameter and is estimated from data.
